# Ultrafast dynamics induced by the interaction of molecules with electromagnetic fields: Several quantum, semiclassical, and classical approaches

**DOI:** 10.1063/1.4996559

**Published:** 2018-01-08

**Authors:** Sergey V. Antipov, Swarnendu Bhattacharyya, Krystel El Hage, Zhen-Hao Xu, Markus Meuwly, Ursula Rothlisberger, Jiří Vaníček

**Affiliations:** 1 Laboratory of Theoretical Physical Chemistry, Institut des Sciences et Ingénierie Chimiques, École Polytechnique Fédérale de Lausanne (EPFL), CH-1015 Lausanne, Switzerland; 2 Laboratory of Computational Chemistry and Biochemistry, École Polytechnique Fédérale de Lausanne (EPFL), CH-1015 Lausanne, Switzerland; 3 Department of Chemistry, University of Basel, Klingelbergstrasse 80, CH-4056 Basel, Switzerland

## Abstract

Several strategies for simulating the ultrafast dynamics of molecules induced by interactions with electromagnetic fields are presented. After a brief overview of the theory of molecule-field interaction, we present several representative examples of quantum, semiclassical, and classical approaches to describe the ultrafast molecular dynamics, including the multiconfiguration time-dependent Hartree method, Bohmian dynamics, local control theory, semiclassical thawed Gaussian approximation, phase averaging, dephasing representation, molecular mechanics with proton transfer, and multipolar force fields. In addition to the general overview, some focus is given to the description of nuclear quantum effects and to the direct dynamics, in which the *ab initio* energies and forces acting on the nuclei are evaluated on the fly. Several practical applications, performed within the framework of the Swiss National Center of Competence in Research “Molecular Ultrafast Science and Technology,” are presented: These include Bohmian dynamics description of the collision of H with H_2_, local control theory applied to the photoinduced ultrafast intramolecular proton transfer, semiclassical evaluation of vibrationally resolved electronic absorption, emission, photoelectron, and time-resolved stimulated emission spectra, infrared spectroscopy of H-bonding systems, and multipolar force fields applications in the condensed phase.

## I. INTRODUCTION

Interaction of molecules with light is the starting point of many remarkable physical and chemical processes occurring on the ultrashort time scales.^1–6^ Due to the microscopic nature of molecules, one might expect that many such processes depend on the quantum nature of both electrons and nuclei, and indeed, this is true in many situations, but fortunately, semiclassical and even classical dynamics of nuclei can provide accurate description of various ultrafast phenomena. Semiclassical and classical dynamics are extremely important for yet another, more pragmatic reason: Exact quantum description requires the solution of the time-dependent Schrödinger equation, which scales exponentially with the number of degrees of freedom, so even on today's supercomputers it is out of reach except for systems with a few atoms. As a result, due to their computational efficiency, classical nuclear dynamics is extremely handy if the only quantum effects are electronic, and semiclassical dynamics—if the nuclear quantum effects can be described approximately. In this article, we will therefore devote some attention to all three approaches for treating nuclear dynamics.

We start with a brief summary of the theory of the interaction of molecules with light (see Sec. II A),^7–9^ including the various approximations made even at the quantum-mechanical level: In particular, we shall always assume the validity of the electric dipole approximation for the interaction potential but allow the fields to be nonperturbative. Then we will discuss, in turn, the time-dependent perturbation theory, valid for weak fields, Condon approximation for the transition dipole, rotating-wave (or quasiresonant), and zero-temperature approximations for electronic transitions, ultrashort-pulse, and separated-pulse approximations for ultrafast laser fields.

The next three sections are devoted to the quantum, semiclassical, and classical treatment of the nuclear dynamics. In all three cases, we give a very brief general overview of the field and then provide a more detailed description of several representative methodologies, which have been either developed or used for applications in the framework of the Swiss National Center of Competence in Research “Molecular Ultrafast Science and Technology” (NCCR MUST). Within quantum dynamics (Sec. II B), these include the multiconfiguration time-dependent Hartree (MCTDH) method,^10,11^ a benchmark for exact quantum dynamics in systems with tens of degrees of freedom, the Bohmian dynamics,^12,13^ a “quantum trajectory”-based method providing an intuitive picture of quantum dynamics, but difficult to implement numerically, and the local control theory (LCT),^14,15^ a very efficient method to reach a control objective, such as increasing a population of a desired electronic state. In Sec. II C on semiclassical dynamics, after a general overview, we present two very simple and complementary methods: the thawed Gaussian approximation (TGA),^16^ based on a single classical trajectory, but requiring the Hessian of the potential energy surface along the trajectory, and the phase averaging/dephasing representation (DR),^8,17,18^ which relies on multiple trajectories, but does not need the Hessian. Finally, in Sec. II D, it is explained how classical force fields can be extended to treat reactive dynamics, involving bond breaking and bond formation, using the molecular mechanics with proton transfer (MMPT),^19^ and how polarizability can be accounted for with multipolar (MTP)^20,21^ force fields. It is also discussed how force fields can be parametrized using a combination of *ab initio* calculations and spectroscopic data. As one of the goals of this paper is to provide a view of the theory of ultrafast dynamics starting from a full quantum, via semiclassical, all the way to purely classical description of the dynamics, we were forced to select only very few but hopefully representative methods from each area.

Section III of this review is devoted to several applications of the methods described in Sec. II. Examples of the quantum methods include the nonadiabatic Bohmian dynamics (NABDY) treatment of the collision of the H atom with the H_2_ molecule and the local control theory applied to the photoinduced ultrafast intramolecular proton transfer in 4-hydroxyacridine (4-HA). Although in principle more general, the local control theory is implemented within the trajectory surface hopping (TSH) method, meaning that the electrons are treated quantum mechanically while the nuclei classically. Applications of semiclassical methods include the on-the-fly *ab initio* semiclassical evaluation of the absorption and photoelectron spectra of ammonia, of the emission spectra of oligothiophenes with up to 105 vibrational degrees of freedom, and the calculations of the time-resolved stimulated emission spectrum of pyrazine with various extensions of phase averaging and dephasing representation. Last but not least, examples of classical molecular dynamics (MD) include the computational infrared spectroscopy of H-bonding systems and the applications of multipolar force fields in the condensed phase, including CO in myoglobin, 1 D- and 2 D-infrared spectroscopy of CN^–^, protein-ligand binding, and vibrational relaxation of solvated CN^–^.

In addition to reviewing examples of quantum, classical, and semiclassical methodologies for simulating ultrafast dynamics, our goal is to point out several emerging notions. On one hand, the term “quantum chemistry” has until rather recently typically evoked the quantum treatment of only the electronic structure. “Molecular dynamics,” on the other hand, typically meant classical nuclear dynamics in empirically parametrized force fields. These restrictions have changed over the years:

First, nuclear quantum effects are important in many fields of chemical physics ranging from spectroscopy to kinetics, and thanks to the improved efficiency of computers these effects are being included in an increasing number of simulations. That is why we devote two sections of this review to quantum and semiclassical dynamics, where the nuclear quantum effects are treated either exactly or approximately.

In many situations, however, even classical molecular dynamics (MD) simulations provide considerable insight. The classical MD is an especially useful tool when the system size becomes large and/or the dynamics beyond the first few picoseconds is important, i.e., situations, in which quantum and semiclassical methods are prohibitively expensive.

With increasing computer power, it has become possible to combine classical MD simulation with electronic structure calculations, which opened the field of *ab initio* MD simulations.^22–24^ Here the forces acting on the nuclei are computed using the *ab initio* electronic structure “on the fly,” i.e., only where they are required during the dynamics. This circumvents the tedious parametrization of force fields and is extremely useful if the problem at hand only requires a single or few trajectories. However, if many trajectories are needed for a statistically significant exploration of phase space,^25,26^ in simulations of very long-time (such as microsecond) dynamics, or in very large systems, where *ab initio* electronic structure remains too costly, it still pays off to construct, once for all, a parametrized force field, which is much cheaper to evaluate repeatedly later. Yet, as the *ab initio* electronic structure becomes gradually more accurate and easier to evaluate, e.g., with the use of graphical processing units,^27–31^ it appears that *ab initio* dynamics will become increasingly practical and popular in the future. That is the reason why we also put an emphasis on the trajectory-based quantum and semiclassical methods, which are naturally suited for the on-the-fly *ab initio* implementation: among the quantum methods, it is the Bohmian dynamics, among the mixed quantum-classical methods, the trajectory surface hopping implementation of the local control theory, and among the semiclassical methods, the thawed Gaussian approximation.

Nonetheless, the reader should be warned about terminology. In this review, we are somewhat casual about what we call “*ab initio*”—in particular, we include density functional theory as a fair game for the electronic structure. To avoid confusion, some authors use the terms “direct dynamics” or “first-principles dynamics” for the same concept. In addition, we include both methods where the *ab initio* or density functional electronic structure is evaluated on the fly, i.e., during the dynamics, and methods where the electronic structure calculations are used to design analytical potential energy surfaces before the actual dynamics.

The applications chapter is followed by a summary, which further reflects on the themes of ultrafast dynamics, nuclear quantum effects, and on-the-fly *ab initio* dynamics, and concludes the paper. For ease of reference, the acronyms used in this article are included in Nomenclature.

## II. THEORY

### A. Interaction of a molecule with electromagnetic field

In this article, we discuss molecular dynamics following either electronic or vibrational excitations induced by the interaction with electromagnetic field. While an electronic excitation can induce nonadiabatic dynamics between different electronic states, and some of the applications below do involve nonadiabatic dynamics, in this section we will focus on adiabatic dynamics, i.e., dynamics on a single electronic potential energy surface, for three reasons: first, nonadiabatic dynamics is discussed much more thoroughly in another article of this special issue,^32^ second, the subject of nonadiabatic dynamics, including spectroscopic detection, conical intersections, and associated geometric phase effects, has been reviewed extensively in the literature,^33–39^ and third, as we show below, many interesting phenomena, even in electronic spectroscopy, depend on dynamics on a single Born-Oppenheimer potential energy surface (or, in time-resolved spectroscopy with well-separated ultrashort pulses, depend on a sequence of such elementary steps, each of which takes place on a single surface).

#### 1. Exact dynamics, electric dipole approximation, and quasiresonant condition

To justify our focus on electronically adiabatic dynamics, we start the discussion with the full molecular wave function that involves both electronic and nuclear degrees of freedom. This will be useful particularly since several applications come from electronic spectroscopy, where the electromagnetic field induces the transition of the molecule to a different electronic state, which is then followed by nuclear adiabatic dynamics on the corresponding, new potential energy surface.

Assuming, for simplicity, only two electronic states, the time-dependent molecular wavepacket can be written as

$$|\psi (t)〉=\sum_{n=1}^{2}|\psi_{n}(t)〉|n〉=\left(\begin{array}{c} \psi_{1}(t)\\ \psi_{2}(t) \end{array}\right),$$ (1)

where $|\psi_{n}(t)〉$ is a time-dependent nuclear wavepacket on the electronic surface *n* and |n〉 the corresponding *n*th electronic state. Evolution of |ψ(t)〉 is given by the time-dependent molecular Schrödinger equation

$$i\hbar \frac{d}{dt}|\psi (t)〉=\hat{H}(t)|\psi (t)〉,$$ (2)

driven by the Hamiltonian

$$\hat{H}(t)={\hat{H}}_{\text{mol}}+{\hat{V}}_{\text{int}}(t),$$ (3)

where ${\hat{H}}_{\text{mol}}$ is the molecular Hamiltonian and ${\hat{V}}_{\text{int}}(t)=-\hat{\vec{\mu }}\cdot \vec{E}(t)$ the interaction potential of the molecule with the electromagnetic field $\vec{E}(t)$ via the electric transition dipole moment $\hat{\vec{\mu }}$. Above, we have introduced compact notation in which the **bold** face denotes the *S*-component vectors in the electronic Hilbert space, such as ψ(t) (*S*, here equal to 2, is the number of relevant electronic states), or *S *×* S* matrices representing electronic operators, whereas the hat $\hat{\;}$ denotes nuclear operators; the arrow $\vec{\;}$ stands for vectors in the ambient, 3-dimensional space. The form of the interaction potential ${\hat{V}}_{\text{int}}(t)$ assumes the validity of the *electric dipole approximation*^40^ but allows rather strong, nonperturbative fields.

In addition, we assume that there are no nonadiabatic or spin-orbit couplings among the two electronic states and the only electronic transitions are induced by the interaction with the electromagnetic field. To rigorously justify neglecting nonadiabatic or spin-orbit couplings, several criteria^32,35,37,38^ can be used, starting from static criteria such as the strength of nonadiabatic couplings or the size of the energy gap between electronic states to more dynamical criteria such as the population dynamics. Among the most rigorous dynamical criteria, “adiabaticity”^41–44^ is defined as the fidelity, i.e., overlap of the adiabatically and nonadiabatically evolved molecular wave functions: If adiabaticity is 1, the nonadiabatic effects can be neglected, whereas if adiabaticity is 0, they must be included in the simulation. In addition, there exist approximate methods to evaluate this adiabaticity efficiently without solving the full Schrödinger equation.^41,43,44^

An infrared laser field will mostly induce vibrational (or rovibrational) transitions and, as a result, one may consider only the diagonal matrix element ${\hat{\vec{\mu }}}_{11}$ of the transition dipole operator, setting the others to zero, and evolve only the wavepacket $|\psi_{1}(t)〉$ on the initial surface. This is true both for weak and strong fields.

In contrast, a visible or ultraviolet laser field will excite electronic transitions, and if it is approximately in resonance with the transition from state 1 to state 2, we are allowed to retain only the off-diagonal elements of the transition dipole moment

$$\hat{\vec{\mu }}\approx \left(\begin{array}{cc} 0 & {\hat{\vec{\mu }}}_{12}\\ {\hat{\vec{\mu }}}_{21} & 0 \end{array}\right).$$ (4)

This is a special case of the *quasiresonant condition*.^45–47^

If the fields are so strong that perturbation theory breaks down, one must treat the electric field explicitly and worry about the coupled dynamics on the two surfaces—in other words, evolve the two-component state |ψ(t)〉. In Sec. II A 2, we show that if perturbation theory is valid, one can think of the electronic transition as instantaneous and evolve the nuclear wavepacket adiabatically on the second surface.

#### 2. Perturbation theory, zero-temperature, and Condon approximations

For sufficiently weak fields or for short interaction times, one may employ the *time-dependent perturbation theory*. Whereas the first-order perturbation theory is often sufficient for linear spectroscopy, the second order is required, e.g., for Raman spectroscopy and the third order (or higher) for more sophisticated nonlinear and time-resolved spectroscopic techniques.^8^ For brevity, in this subsection, we only consider the first-order perturbation theory, within which the molecular state evolves as

$$|\psi (t)〉={\hat{U}}_{\text{mol}}(t)|\psi (0)〉-\frac{i}{\hbar }\int_{-\infty }^{t}dt^{\prime }{\hat{U}}_{\text{mol}}(t-t^{\prime }){\hat{V}}_{\text{int}}(t^{\prime }){\hat{U}}_{\text{mol}}(t^{\prime })|\psi (0)〉,$$ (5)

where ${\hat{U}}_{\text{mol}}(t)=\text{exp}\;(-i{\hat{H}}_{\text{mol}}t/\hbar )$ denotes the molecular evolution operator in the absence of the electric field.

For vibrational transitions, Eq. (5) simplifies as

$$|\psi_{1}(t)〉={\hat{U}}_{1}(t)|\psi_{1}(0)〉+\frac{i}{\hbar }\int_{-\infty }^{t}dt^{\prime }{\hat{U}}_{1}(t-t^{\prime }){\hat{\vec{\mu }}}_{11}\cdot \vec{E}(t^{\prime }){\hat{U}}_{1}(t^{\prime })|\psi_{1}(0)〉,$$ (6)

where ${\hat{U}}_{n}(t)=\text{exp}\;(-i{\hat{H}}_{n}t/\hbar )$ stands for the nuclear evolution operator in the electronic state *n*. Note that the second electronic state plays no role whatsoever. The molecule first evolves independently of the field up to time $t^{\prime }$, when it feels the field instantaneously, resulting in rovibrational transitions, and then evolves up to time *t*, again with the molecular Hamiltonian only. This is integrated over all possible interaction times $t^{\prime }$. Equation (6) provides a simple, yet rigorous criterion for the validity of the time-dependent perturbation theory, namely, the second term in Eq. (6), which is due to the interaction with the field, must be smaller than the first term, due to the molecular evolution in the absence of the field. Since the first term describes a unitary evolution and, therefore, has a unit norm at all times, the criterion for the validity of the perturbation theory is that the norm of the second term be smaller than 1. In contrast to time-independent perturbation theory, where time plays no role, time-dependent perturbation theory breaks down not only with increasing magnitude of the electric field $\vec{E}(t)$, but also with increasing interaction times (i.e., longer pulses of the same strength).^9^

For electronic transitions, expression (5) also simplifies, but in a different way. The only interesting part is the first-order term describing the wavepacket generated by the field on the second electronic surface

$$|\psi_{2}(t)〉=\frac{i}{\hbar }\int_{-\infty }^{t}dt^{\prime }{\hat{U}}_{2}(t-t^{\prime }){\hat{\vec{\mu }}}_{21}\cdot \vec{E}(t^{\prime }){\hat{U}}_{1}(t^{\prime })|\psi_{1}(0)〉.$$ (7)

This equation implies that the initial state first evolves freely on the first surface, then, at time $t^{\prime }$, interacts with the field, which induces instantaneously an electronic transition to the second electronic state, and, finally, evolves for the remaining time on the second electronic surface. Again, this is integrated over all possible interaction times $t^{\prime }$. [Here we assumed no initial occupation of the second electronic state; hence, the perturbation theory is valid if $\left|\left|\psi_{2}(t\right)\right||\ll 1$, which can be expressed approximately by requiring that $\hbar^{-1}\int_{-\infty }^{t}{\vec{\mu }}_{21,\text{av}}\cdot {\vec{E}}_{\text{env}}(t^{\prime })\;dt^{\prime }\ll 1$, where ${\vec{\mu }}_{21,\text{av}}$ is the average of the transition dipole over the molecular wavepacket (or its constant value within the Condon approximation) and ${\vec{E}}_{\text{env}}(t^{\prime })$ the slowly varying envelope of the electric field (equivalently, within a factor of 2, the electric field in the so-called rotating frame).]

At room temperature, most of the molecules are typically in the vibrational ground state of the initial electronic state, which is, in particular, an eigenstate of ${\hat{H}}_{1}$; hence, the first evolution operator ${\hat{U}}_{1}(t^{\prime })$ yields only a phase factor $\text{exp}\;(-iE_{1,0}t/\hbar )$, which results in an overall shift of an electronic spectrum by the zero point vibrational energy $E_{1,0}$ of the initial electronic state. As a result, in the case of electronic transitions, the only interesting dynamics occurs after time $t^{\prime }$, in the second electronic state, and hence, as promised, the problem reduces to adiabatic dynamics on the second surface. The assumption that the initial state is a vibrational ground state of ${\hat{H}}_{1}$ is usually referred to as the *zero-temperature approximation* and avoids the necessity of Boltzmann averaging over different initial states. It is a good approximation for vibrationally resolved electronic spectroscopy.

If electronic transitions are of interest, one also frequently makes the *Condon approximation*,^48–50^ which amounts to ignoring the dependence of the transition dipole on the nuclear coordinates: ${\hat{\vec{\mu }}}_{12}\approx \text{const}={\vec{\mu }}_{12}$. Note that removing the hat from ${\hat{\vec{\mu }}}_{12}$ permits taking the transition dipole in front of the integral sign in Eq. (7). In contrast, an analogous approximation is not useful for vibrational transitions. If the dipole moment ${\hat{\vec{\mu }}}_{11}$ were independent of nuclear coordinates, Eq. (6) would yield a boring result $|\psi_{1}(t)〉=\left[1+(i/\hbar )\int_{-\infty }^{t}dt^{\prime }{\vec{\mu }}_{11}\cdot \vec{E}(t^{\prime })\right]{\hat{U}}_{1}(t)|\psi_{1}(0)〉$, i.e., the field would only add an overall phase to the field-free evolution of the initial state. In particular, no vibrational transitions would occur.

#### 3. Adiabatic quantum, semiclassical, and classical dynamics

To summarize, we have considered either weak or strong electromagnetic fields inducing either vibrational or electronic transitions and showed that only in the case of strong fields resonant with electronic transitions, one has to perform explicitly electronically nonadiabatic dynamics (we will show an example of this when discussing local control theory in Secs. II B 3 and III A 2). In the three other cases, the dynamics is adiabatic and depends only on the molecular Hamiltonian if the fields are weak (both infrared and visible to ultraviolet light, see Secs. II C 2, III B 2, III B 3, and III C 2), or, on the full time-dependent Hamiltonian if the fields are infrared and strong.

It is worth mentioning that there exist several powerful, nonperturbative approaches to molecular quantum dynamics that sometimes can be even more efficient than perturbation theory, especially if higher order perturbation theory is required. Among these, it is worth mentioning the work of Seidner, Stock, and Domcke^33,51–53^ who also provide an elegant way to analyze nonperturbative calculations of ultrafast spectra which allow extracting individual spectroscopic signals, resolved in time, frequency, and direction of emission, from the total polarization, and more recent work of Gelin, Egorova, and Domcke^54–57^ who proposed a time-domain spectroscopic technique based on strong pump and probe pulses to access temporal resolution that is not limited by the pulse duration and that cannot be obtained by weak pump and probe pulses.

When perturbation theory is sufficient, similar simplifications to those discussed above in detail for the first-order perturbation theory occur also in nonlinear, pump-probe spectroscopy if the pulses are, in addition, *ultrashort* (i.e., long on the electronic dephasing time scale and short on nuclear time scale) and *well separated*.^8,34,58–63^ Although higher order perturbation theory is required, for weak and well-separated ultrashort pulses, one can compute, e.g., transient absorption or time-resolved stimulated emission spectra within the “doorway/window” picture,^58^ in which the interaction with the probe pulse can be treated simply as the first-order spectroscopy of a nonstationary state prepared by the pump pulse.^59,60^ The calculation is done by performing a sequence of “elementary” adiabatic dynamics steps on different surfaces with instantaneous switches in between (see Secs. II C 1 and III B 1). In the following, we will describe various approaches to perform this elementary, adiabatic quantum dynamics on a single electronic surface, which can be stated as the problem of solving one of the following time-dependent Schrödinger equations for nuclei:

$$\begin{array}{c} i\hbar \frac{d}{dt}|\psi (t)〉=\hat{H}|\psi (t)〉\;\text{or}\;i\hbar \frac{d}{dt}|\psi (t)〉=\left[\hat{H}-\hat{\vec{\mu }}\cdot \vec{E}(t)\right]|\psi (t)〉. \end{array}$$ (8)

For simplicity, we dropped the electronic state indices on the Hamiltonian, state and the transition dipole since they are no longer needed. The former equation applies for perturbative fields, while the latter is needed for strong infrared fields.

In particular, we will discuss various approaches with different degrees of accuracy of the treatment of electronic structure [i.e., accuracy of $\hat{H}$ and $\hat{\vec{\mu }}$ in Eq. (8)] and of the nuclear quantum dynamics [i.e., accuracy of |ψ(t)〉, given $\hat{H}$ and $\hat{\vec{\mu }}$]. We start with the in-principle exact quantum dynamics methods whose only approximation consists in the numerical implementation. Then we consider *ab initio* semiclassical dynamics, an extension of *ab initio* classical dynamics that takes into account quantum interference by carrying semiclassical phase information along the classical trajectories and provides an intuitive understanding of the dynamics. Finally, we discuss classical molecular dynamics using reactive, multipolar, and *ab initio*-based force fields, an approach, which, by replacing the wave function evolution by classical trajectories, permits to treat the largest systems and can be remarkably accurate in cases where nuclear quantum effects are not important.

### B. Quantum dynamics

The motion of the nuclei in a molecule, which is inherently quantum mechanical in nature, can be described most accurately by the solution of the time-dependent Schrödinger equation.^9^ For a time-independent molecular Hamiltonian, the knowledge of a nuclear wavepacket at all times carries essentially the same information as that provided by solving the time-independent Schrödinger equation and knowing all the eigenstates of the Hamiltonian. It is the particular problem at hand which makes adopting one approach over the other preferable. For example, low-resolution electronic absorption or photoelectron spectra typically depend on a rather short-time behavior of the system, making the time-dependent perspective the obvious choice. For time-dependent Hamiltonians, which do not have well-defined eigenstates, the time-dependent approach is even more important since the time-independent approach cannot be used at all.

The numerical solution of the time-dependent Schrödinger equation relies on a suitable discretization of the wave function as well as the Hamiltonian, typically in terms of a set of basis functions or grid points, and on a numerical algorithm chosen to propagate the initial wave function in time. Over the years, many numerical propagation schemes have been developed and the detailed description of various approaches can be found in specialized reviews.^64–71^ The most popular propagation methods include, e.g., the Chebyshev^72^ and iterative Lanczos propagators,^73–75^ both of which employ an expansion of the action of the time-evolution operator into a convergent series. Other widely used methods, based on the explicit integration of the differential equation, include the finite differences^76–78^ and Runge Kutta^79,80^ schemes.

Among numerical approaches that take into account the geometric structure of the time-dependent Schrödinger equation, i.e., which preserve the time-reversal symmetry, unitarity, and symplectic structure of the quantum dynamics exactly, the one most commonly used is the split-operator method,^81,82^ which takes advantage of treating the kinetic and potential energy operators in their natural representations (i.e., momentum and coordinate representations, respectively), in which the relevant operators are represented by diagonal matrices. Originally formulated for the second-order splitting, the algorithm has been extended to an arbitrary order of accuracy in the time step.^83–87^ Recently, the split-operator method was combined with the Magnus expansion^88–93^ to construct geometric integrators of arbitrary order of accuracy in the time step not only for the exact treatment of the interaction of the molecule with electromagnetic field but also for an arbitrary combination of the Condon, rotating wave, and ultrashort pulse approximations.^94,95^

All of the above mentioned numerical propagation methods have their merits, and their performance depends on the particular problem under consideration. However, the number of basis functions or grid points needed to represent a wave function typically increases exponentially with the number of degrees of freedom considered, which makes the numerically exact quantum dynamical calculations practically impossible for systems with large dimensionality. This is the main reason behind the long-standing search for approximate but numerically efficient methods to solve the time-dependent Schrödinger equation.

#### 1. Multi-configuration time-dependent Hartree (MCTDH) method

The time-dependent Hartree method serves as a way to circumvent the exponential-scaling problem, where the molecular wave function is represented as a Hartree-product of one-dimensional *time-dependent* basis functions, known as *single-particle functions* (SPFs). In spite of its appealing simplicity and computational efficiency, however, this method suffers from lack of accuracy. Being a single reference ansatz, it neglects a large part of the correlation present between different degrees of freedom.^96^ The multi-configuration time-dependent Hartree (MCTDH) approach emerged as a natural extension of the time-dependent Hartree method, where the molecular wave function is expanded in terms of several Hartree products/configurations.^10,97,98^ The MCTDH method can be viewed as a trade-off between the efficiency of the time-dependent Hartree method and the accuracy of a numerically exact treatment (analogous to full configuration integration in electronic-structure theory).^10,11,99^ The high flexibility in choosing the number of SPFs opens the way to access the full range of approximations between time-dependent Hartree and the numerically exact solution. As the time-dependent SPFs closely follow the time evolution of the nuclear wavepacket, convergence can be achieved relatively easily.

In MCTDH, the wave function is defined by the following ansatz:^10,11,99^

$$\begin{array}{c} \psi (Q,t)=\sum_{\gamma_{1}=1}^{n_{1}}\cdots \sum_{\gamma_{D}=1}^{n_{D}}A_{\gamma_{1}\cdots \gamma_{D}}(t)\prod_{\alpha =1}^{D}\varphi_{\gamma_{\alpha }}^{\left(\alpha \right)}(Q_{\alpha },t)=\sum_{\Gamma }A_{\Gamma }\Phi_{\Gamma } \end{array},$$ (9)

where *D* denotes the number of degrees of freedom, *Q* is the vector containing the set of nuclear coordinates, $A_{\gamma_{1}\cdots \gamma_{D}}$ denote the MCTDH expansion coefficients, and $\varphi_{\gamma_{\alpha }}^{\left(\alpha \right)}$ are the $n_{\alpha }$ time-dependent expansion functions (SPFs) for each degree of freedom *α*. $\Phi_{\Gamma }$ is the *D*-dimensional Hartree product of the SPFs represented by the composite index $\Gamma =(\gamma_{1},\ldots ,\gamma_{D})$. For practical purposes, the SPFs have to be represented in terms of an underlying time-independent primitive basis set

$$\varphi_{\gamma_{\alpha }}^{\left(\alpha \right)}(Q_{\alpha },t)=\sum_{\lambda =1}^{N_{\alpha }}c_{\lambda \gamma_{\alpha }}^{\left(\alpha \right)}(t)\chi_{\lambda }^{\left(\alpha \right)}(Q_{\alpha }).$$ (10)

The primitive basis functions are often replaced by a *discrete variable representation* grid.

The SPFs and the time-dependent expansion coefficients in Eq. (9) are determined by variationally solving the time-dependent Schrödinger equation using the *Dirac-Frenkel variational principle*^100,101^

$$〈\delta \psi |\hat{H}-i\frac{\partial }{\partial t}|\psi 〉=0.$$ (11)

After some algebra, one obtains two coupled differential equations for the SPFs and the expansion coefficients

$$i{\dot{A}}_{\Gamma }=\sum_{\Lambda }〈\Phi_{\Gamma }|\hat{H}|\Phi_{\Lambda }〉A_{\Lambda },$$ (12)

$$i|{\dot{\varphi }}_{\gamma }^{\left(\alpha \right)}〉=\sum_{\lambda ,\xi }\left(1-{\hat{P}}^{\left(\alpha \right)}\right){(\rho^{{\left(\alpha \right)}^{-1}})}_{\gamma \lambda }{〈\hat{H}〉}_{\lambda \xi }^{\left(\alpha \right)}|\varphi_{\xi }^{\left(\alpha \right)}〉,$$ (13)

where $P^{\left(\alpha \right)}$ denotes the projection operator on the space spanned by the SPFs for the *α*th degree of freedom, $\rho_{\gamma \lambda }^{\left(\alpha \right)}$ denotes a density matrix, and ${〈\hat{H}〉}_{\lambda \xi }^{\left(\alpha \right)}$ is a matrix of mean-fields.

While in a standard wavepacket propagation *N^D^* numbers are needed to represent a wave function, the memory required to represent an MCTDH wave function is

$$\text{memory}∼n^{D}+DnN,$$ (14)

which is a huge memory saving especially for high-dimensional systems.^102^

The MCTDH ansatz needs to be extended to describe nonadiabatic dynamics. A particularly convenient way is to use the so-called *multi-set* formulation, which employs different sets of SPFs for different electronic states. In this ansatz, the wave function is expanded in the set {|j〉} of diabatic electronic states^103^

$$\psi (Q,t)=\sum_{j=1}^{S}\psi_{j}(Q,t)|j〉,$$ (15)

where the component $\psi_{j}(Q,t)$ is the nuclear wavepacket evolving on the electronic state |j〉 and is represented in the usual MCTDH form as in Eq. (9).

During the last years, MCTDH was used to study different aspects of the nuclear dynamics of molecules and clusters within the NCCR MUST.^104–106^

#### 2. Bohmian dynamics

Despite the overwhelming success of MCTDH in performing accurate quantum dynamical calculations for relatively large systems, it still suffers from an exponential scaling behavior. On the other hand, with most of the existing trajectory based solutions, it is possible to deal with a large number of degrees of freedom, however, at the expense of accuracy. For example, the nuclear trajectory obtained in Ehrenfest dynamics, which typically lies on the mean-field potential, does not have a clear physical meaning. While the widely used trajectory surface hopping (TSH) schemes have been successful in describing some nuclear quantum effects like the branching of nuclear wavepackets, however, by virtue of being classical by construction, it is unable to describe some other quantum phenomena like decoherence and tunneling.

One possible solution to this problem, that is, to achieve an accurate and efficient quantum propagation scheme for the nuclei is to employ the so-called *quantum trajectory* based methods developed by Wyatt *et al.*^12,13^ Having Bohmian (or hydrodynamical) interpretation of quantum mechanics^107,108^ as its backbone, this method provides formally exact equations of motion for quantum trajectories (also known as *fluid elements*), which in principle reproduce the full nuclear wavepacket dynamics. In this class of methods, the complex nuclear wave function is represented in its polar form. The Madelung ansatz ψ(Q,t)=A(Q,t) exp (iS(Q,t)/ℏ) is inserted into the time-dependent Schrödinger equation; separating the real and imaginary parts yields equations of motion for the amplitude and phase of the nuclear wavepacket. The equation for the amplitude is equivalent to a continuity equation for the probability density $\rho =A^{2}$, while the equation for the phase can be interpreted as an extended Hamilton-Jacobi equation

$$-\frac{\partial S}{\partial t}=\frac{1}{2m}{\left(\nabla S\right)}^{2}+V-\frac{\hbar^{2}}{2m}\frac{\nabla^{2}A}{A},$$ (16)

in which

$$Q=-\frac{\hbar^{2}}{2m}\frac{\nabla^{2}A}{A}$$ (17)

is the so-called quantum potential. Together, the continuity and extended Hamilton-Jacobi equations provide a link between this formulation of quantum mechanics and continuum hydrodynamics. This link, in turn, makes it possible to derive a Newton-like equation for the propagation of the fluid elements, giving rise to a swarm of correlated trajectories by virtue of the presence of the quantum potential.

In a recent version of Bohmian mechanics, *ρ* was obtained from kernel density estimation, a concept borrowed from statistics, which is a non-parametric procedure to estimate the probability density function from a finite number of samples. Using such a formulation, it was shown that tunneling probabilities can be readily and accurately computed for 1- and 2-dimensional problems whereas interference effects are oversmoothed.^109^

During the last decade, there has been a constant endeavour to extend the original ideas of *quantum trajectory* based methods in order to be able to apply them to the cases of nuclear dynamics involving more than one electronic state.^110–112^ The specific implementations differ mainly by the way the electronic wave function is represented, namely, the adiabatic or the diabatic representation.^113,114^ It is worth mentioning here that, in spite of being quite promising, these non-adiabatic dynamics schemes suffer quite often from severe computational difficulties. One of the major problems is related to the instability associated with the numerical computation of the quantum potential.

Recently, a scheme has been developed with an aim to solve the non-relativistic high-dimensional quantum dynamics of nuclei and electrons within the framework of Bohmian dynamics employing an adiabatic representation of the electronic states. This method, NABDY (Nonadiabatic Bohmian DYnamics), an on-the-fly trajectory based nonadiabatic molecular dynamics algorithm, is able to capture the nuclear quantum effects which were missing in TSH due to the independent trajectory approximation.^115–117^ This method has been implemented within the CPMD package,^118^ where the electronic energies, classical forces, and the nonadiabatic coupling elements are calculated on-the-fly for each configuration at the DFT/TDDFT level of theory.

For the formal derivation, one can start from the time-dependent Schrödinger equation for the molecular system

$$\hat{H}|\psi (Q,t)〉=i\hbar \frac{\partial }{\partial t}|\psi (Q,t)〉,$$ (18)

where the molecular wave function, |ψ(Q,t)〉, can be expressed in the Born-Huang ansatz as

$$|\psi (Q,t)〉=\sum_{j=1}^{\infty }\psi_{j}(Q,t)|j〉.$$ (19)

Here, the expansion coefficients, $\psi_{j}(Q,t)$, can be interpreted as the nuclear wave function associated with the electronic state |j〉. If expressed in polar form, the complex nuclear wave function reads

$$\psi_{j}(Q,t)=A_{j}(Q,t)\;\text{exp}\left[\frac{i}{\hbar }S_{j}(Q,t)\right].$$ (20)

Inserting Eq. (19) in Eq. (18) and using Eq. (20), we obtain the following equations of motion for the amplitude $A_{j}(Q,t)$ and for the phase $S_{j}(Q,t)$:

$$\begin{array}{c} \frac{\partial A_{j}(Q,t)}{\partial t}=-\sum_{\gamma }\frac{1}{M_{\gamma }}\nabla_{\gamma }A_{j}(Q,t)\nabla_{\gamma }S_{j}(Q,t)-\sum_{\gamma }\frac{1}{2M_{\gamma }}A_{j}(Q,t)\nabla_{\gamma }^{2}S_{j}(Q,t)\\ +\sum_{\gamma i}\frac{\hbar }{2M_{\gamma }}D_{ji}^{\gamma }(Q)A_{i}(Q,t)\text{Im}\left[e^{i\phi (Q,t)}\right]\\ -\sum_{\gamma ,i\neq j}\frac{\hbar }{M_{\gamma }}d_{ji}^{\gamma }(Q)\nabla_{\gamma }A_{i}(Q,t)\text{Im}\left[e^{i\phi (Q,t)}\right]\\ -\sum_{\gamma ,i\neq j}\frac{1}{M_{\gamma }}d_{ji}^{\gamma }(Q)A_{i}(Q,t)\nabla_{\gamma }S_{i}(Q,t)\text{Re}\left[e^{i\phi (Q,t)}\right] \end{array}$$

and

$$\begin{array}{c} -\frac{\partial S_{j}(Q,t)}{\partial t}=\sum_{\gamma }\frac{1}{2M_{\gamma }}{\left(\nabla_{\gamma }S_{j}(Q,t)\right)}^{2}+E_{j}^{el}(Q)-\sum_{\gamma }\frac{\hbar^{2}}{2M_{\gamma }}\frac{\nabla_{\gamma }^{2}A_{j}(Q,t)}{A_{j}(Q,t)}\\ +\sum_{\gamma i}\frac{\hbar^{2}}{2M_{\gamma }}D_{ji}^{\gamma }(Q)\frac{A_{i}(Q,t)}{A_{j}(Q,t)}\text{Re}\left[e^{i\phi (Q,t)}\right]\\ -\sum_{\gamma ,i\neq j}\frac{\hbar^{2}}{M_{\gamma }}d_{ji}^{\gamma }(Q)\frac{\nabla_{\gamma }A_{i}(Q,t)}{A_{j}(Q,t)}\text{Re}\left[e^{i\phi (Q,t)}\right]\\ +\sum_{\gamma ,i\neq j}\frac{\hbar }{M_{\gamma }}d_{ji}^{\gamma }(Q)\frac{A_{i}(Q,t)}{A_{j}(Q,t)}\nabla_{\gamma }S_{i}(Q,t)\text{Im}[e^{i\phi (Q,t)}], \end{array}$$

where

$$\phi (Q,t)=\frac{1}{\hbar }\left[S_{i}(Q,t)-S_{j}(Q,t)\right],$$ (21)

$$H_{ji}(Q)=〈j|{\hat{H}}_{el}|i〉.$$ (22)

The first-order ($d_{ji}^{\gamma }$) and the second-order ($D_{ji}^{\gamma }$) nonadiabatic couplings are, respectively,

$$d_{ji}^{\gamma }=〈j|\nabla_{\gamma }|i〉,$$ (23)

$$D_{ji}^{\gamma }=〈j|\nabla_{\gamma }^{2}|i〉.$$ (24)

In the Hamilton-Jacobi formulation of mechanics, the phase of the nuclear wavepacket can be related to its momentum as

$$\nabla_{\beta }S_{j}(Q,t)=P_{\beta }^{j},$$ (25)

which gives rise to a Newton-like equation for the motion of the nuclei

$$M_{\beta }\frac{d^{2}Q_{\beta }}{{\left(dt^{j}\right)}^{2}}=-\nabla_{\beta }\left[E_{j}^{el}(Q)+Q_{j}(Q,t)+D_{j}(Q,t)\right],$$ (26)

where the definition of the time-derivative in the Lagrangian frame has been employed

$$\frac{d}{dt^{j}}=\frac{\partial }{\partial t}+\sum_{\gamma }\nabla_{\gamma }\frac{S_{j}(Q,t)}{M_{\gamma }}\cdot \nabla_{\gamma }.$$ (27)

It is clear from Eq. (26) that in addition to the usual classical potential, $E_{j}^{el}(Q)$, the nuclei will feel a quantum potential $Q_{j}(Q,t)$ as well, which induces adiabatic nuclear quantum effects. $D_{j}(Q,t)$ is the nonadiabatic quantum potential and is responsible for electronic interstate couplings.

Numerically, a conventional quantum trajectory propagation scheme is used to start the adiabatic dynamics of an initial wavepacket represented as an ensemble of fluid elements on a single electronic state. The nonadiabatic couplings are constantly monitored during the adiabatic propagation of the quantum trajectories. When their strengths exceed a pre-defined threshold, the algorithm starts the dynamics on the coupled electronic states.

#### 3. Local control theory with *ab initio* molecular dynamics: A computationally efficient scheme to achieve control

Since a couple of decades, ultrafast laser pulses have increasingly been employed to induce certain dynamical events in molecules leading to the emergence of fields such as femtochemistry and femtobiology.^119–121^ The shaping of laser pulses to control chemical reactions has been a long-standing topic of interest for both theorists and experimentalists.^122–126^ The term *coherent control* of chemical reactions grossly includes all those schemes which optimize an external radiation field such that it can induce a transition from an initial state to a final state (also called *target*) after a certain time. The most well-known ones are the pioneering success of the Tannor-Rice-Kosloff pump-dump scheme^14,127^ and the Brumer-Shapiro scheme.^128^

One of the most commonly employed coherent control techniques is *optimal control theory*.^129^ This is, in general, a global control scheme, where the control field is constructed variationally through an iterative process of forward-backward propagations considering the information of the entire dynamics of the system. This scheme carries many similarities with the experimental learning algorithm approach.^130^ Despite its apparent success, it has a few significant disadvantages. One of the major problems is the computational expense it demands due to the involvement of multiple forward-backward propagations. Another practical drawback is the fact that, in spite of giving the optimized pulse producing the desired target, optimal control theory does not provide direct information leading to a detailed understanding of the underlying mechanism which often requires further analysis.^131^

Unlike optimal control theory, local control theory (LCT) departs from the global picture and calculates the field on-the-fly taking into account the instantaneous response of the system dynamics. In LCT, one typically calculates the field at each time step to ensure the increase/decrease in the expectation value of an operator of interest, such as an electronic state population, vibrational state population, or nuclear momentum.^15,124–126,132,133^ Being computationally much faster than optimal control theory, and being more flexible, LCT is widely considered as the method of choice to achieve coherent control of larger systems. It should be mentioned here that a connection between optimal control theory and LCT can be established by considering the fact that, at least in some cases, LCT equations can be derived by solving the optimal control theory equations employing Krotov's scheme.^15^

The Hamiltonian of a molecular system, upon interaction with a radiation field, can be written as

$$\hat{H}={\hat{H}}_{\text{mol}}+{\hat{V}}_{\text{int}},$$ (28)

where ${\hat{H}}_{\text{mol}}$ is the field-free Hamiltonian of the system and ${\hat{V}}_{\text{int}}$ describes the interaction of the system with the electromagnetic field. In the dipole approximation, the interaction part of the Hamiltonian can conveniently be expressed as

$${\hat{V}}_{\text{int}}^{ji}=-{\hat{\vec{\mu }}}_{ji}\cdot \vec{E}(t).$$ (29)

The main objective of LCT is to calculate an electric field on-the-fly, at each time step, as a response of the instantaneous dynamics of the system to ensure the increase (or decrease) of the expectation value of some predefined operator. If we consider the time evolution of an arbitrary operator $\hat{O}$, one finds

$$\begin{array}{c} \frac{d〈\hat{O}〉}{dt}=\frac{i}{\hbar }〈\psi (t)|[{\hat{H}}_{\text{mol}},\hat{O}]|\psi (t)〉+\frac{i}{\hbar }〈\psi (t)|[{\hat{V}}_{\text{int}},\hat{O}]|\psi (t)〉, \end{array}$$ (30)

where |ψ(t)〉 is the molecular state vector at time *t*. This equation shows that if $\hat{O}$ and ${\hat{V}}_{\text{int}}$ do not commute, it is possible to shape the external field to influence a desired change in the expectation value of $\hat{O}$. Assuming that ${\hat{H}}_{\text{mol}}$ commutes with $\hat{O}$, which is the case only in the absence of nonadiabatic couplings, Eq. (30) can be written as

$$\frac{d〈\hat{O}〉}{dt}=-\frac{i}{\hbar }\vec{E}(t)〈\psi (t)|[\hat{\vec{\mu }},\hat{O}]|\psi (t)〉,$$ (31)

and therefore, the desired control may be achieved by changing the temporal evolution of $\vec{E}$. If we consider the transfer of electronic population to the state |i〉, the corresponding operator to be employed is the projector $P_{i}=|i〉〈i|$. In the absence of nonadiabatic couplings, the time evolution of the projector operator can then be written as

$$\frac{d〈P_{i}〉}{dt}=-\frac{i}{\hbar }\vec{E}(t)〈\psi (t)|[\hat{\vec{\mu }},P_{i}]|\psi (t)〉.$$ (32)

Equation (32) is common to most of the LCT implementations irrespective of the underlying dynamical method. However, in the method developed in the framework of the NCCR MUST, LCT has been implemented within a trajectory surface hopping (TSH) *ab initio* molecular dynamics scheme.^134^ All the required quantities, such as electronic energies, nuclear forces, nonadiabatic couplings, and transition dipole elements, have been calculated on-the-fly with LR-TDDFT as implemented in the software package CPMD. Within the TSH ansatz, the total wave function for trajectory *α* is approximated as

$$|\psi^{\alpha }(t)〉=\sum_{j=1}^{\infty }C_{j}^{\alpha }(t)|j〉,$$ (33)

where the complex-valued time-dependent amplitude $C_{j}^{\alpha }(t)$ substitutes the nuclear wavepacket in the corresponding quantum-dynamical ansatz and apportions trajectories among electronic states. Applying this ansatz to Eq. (32) and expanding the projector operator for trajectory *α*, it is straightforward to get the following equation:

$$\frac{d〈P_{i}^{\alpha }〉}{dt}=-2{\vec{E}}^{\alpha }(t)\sum_{j}\text{Im}[C_{j}^{\alpha *}(t){\hat{\vec{\mu }}}_{ji}C_{i}^{\alpha }(t)].$$ (34)

From this equation, it is clear that choosing the electric field to be

$${\vec{E}}^{\alpha }(t)=\pm \lambda \sum_{j}\text{Im}[C_{j}^{\alpha *}(t)C_{i}^{\alpha }(t){\hat{\vec{\mu }}}_{ji}]$$ (35)

ensures, depending on the sign, that d〈P(t)〉/dt increases or decreases at all times. LCT has been applied to a photoinduced intramolecular proton transfer reaction, which is described in more detail in Sec. III.

### C. Semiclassical dynamics

As mentioned above, a direct solution of the time-dependent Schrödinger equation for large systems is unfeasible due to the exponential scaling of the computational cost with the number of dimensions. Moreover, the exact quantum dynamics requires construction of global potential energy surfaces, which is a daunting task by itself.

In this respect, semiclassical trajectory-based methods provide a powerful tool for molecular dynamics simulations. On one hand, as in the Bohmian or *ab initio* classical dynamics, the propagation of classical trajectories requires only a local knowledge of the potential energy surface, allowing on-the-fly evaluation of necessary *ab initio* data. On the other hand and in contrast to classical dynamics simulations, semiclassical methods can approximately describe nuclear quantum effects, such as the zero-point energy and quantum coherences, by virtue of the phase carried along each trajectory.

In particular, the *Herman–Kluk* initial value representation,^135–137^ which stems from the stationary-phase approximation to the Feynman path integral propagator, has been successfully merged with on-the-fly dynamics to calculate vibrationally resolved spectra^138–141^ and internal conversion rates.^142^ Within the Herman–Kluk approximation, the quantum evolution operator can be written as

$$e^{-i\hat{H}t/\hbar }\approx h^{-D}\int dq_{0}dp_{0}\;R_{t}(q_{0},p_{0})e^{iS_{t}(q_{0},p_{0})/\hbar }|q_{t}p_{t}〉〈q_{0}p_{0}|,$$ (36)

where *D* is the number of degrees of freedom in the system, $\left(q_{t},p_{t}\right)$ denote the phase-space coordinates at time *t* of a point along the classical trajectory, and $S_{t}(q_{0},p_{0})$ is the corresponding classical action. In the position representation, the wave functions of the coherent states from Eq. (36) are given by

$$〈r|qp〉={\left(\frac{\det g}{\pi^{D}}\right)}^{1/4}\;\text{exp}\;\left[-\frac{1}{2}{\left(r-q\right)}^{T}\cdot g\cdot (r-q)+\frac{i}{\hbar }p^{T}\cdot (r-q)\right],$$ (37)

where *g* is the coherent state width matrix and the Herman–Kluk prefactor is given by

$$R_{t}(q_{0},p_{0})=\sqrt{\det\left[\frac{1}{2}\left(M_{qq}+g^{-1}\cdot M_{pp}\cdot g-i\hbar M_{qp}\cdot g+\frac{i}{\hbar }g^{-1}\cdot M_{pq}\right)\right]}$$ (38)

with $M_{\alpha \beta }=\partial \alpha_{t}/\partial \beta_{0}$ being the components of the stability (monodromy) matrix. The phase-space integral in Eq. (36) is usually evaluated by sampling the initial conditions of classical trajectories using Monte Carlo techniques; the subsequent propagation requires computing the potential energy to evolve the action *S*, forces to evolve positions and momenta, and the Hessian to evolve the stability matrix *M*.

Despite some progress, the straightforward application of the Herman–Kluk initial value representation to systems with many degrees of freedom is limited. The oscillatory nature of the integrand in Eq. (36) requires a very large number of trajectories to converge the results, which, together with the expensive Hessian calculations, keeps the overall computational cost high. The computational burden can be partially alleviated by invoking additional approximations such as a prefactor-free propagator,^143^ time averaging,^144^ and Filinov filtering (cellularization).^145–147^ The latter technique has been widely used to improve Monte Carlo statistics^147–149^ and to derive new approximate semiclassical methods.^149–151^ The time-averaging has proved to be particularly useful in the context of on-the-fly simulations as a central ingredient of the multiple-coherent-states time-averaged semiclassical initial value representation.^139,140,152^ This method is especially well suited for the determination of vibrational frequencies and prediction of vibrational spectra.^152–155^

#### 1. Phase averaging, dephasing representation, and extensions

Within the domain of validity of perturbation theory, all dynamical phenomena in complex systems can be described in terms of time correlation functions. For example, in the time-dependent approach, pioneered by Heller,^156^ many linear and nonlinear spectra of a molecule can be obtained via the Fourier transform of an appropriate wavepacket correlation function. Thus, many semiclassical dynamics methods are specifically designed to approximate directly the correlation function rather than the solution of the time-dependent Schrödinger equation itself.

Methods employing the correlations functions invoke the time-dependent perturbation theory, where the dynamics involves only the molecular Hamiltonian, which is time-independent. Let us, therefore, consider a general wave packet correlation function (sometimes called the fidelity amplitude^157,158^) given by

$$f(t)=〈\psi_{1}(t)|\psi_{2}(t)〉=〈\psi |e^{i{\hat{H}}_{1}t/\hbar }e^{-i{\hat{H}}_{2}t/\hbar }|\psi 〉,$$ (39)

where ${\hat{H}}_{1}$ and ${\hat{H}}_{2}$ are the Hamiltonian operators corresponding to different electronic states of the system and |ψ〉 is the initial state, which is typically an eigenstate of ${\hat{H}}_{1}$ or ${\hat{H}}_{2}$. A remarkably simple approximation for Eq. (39) is given by the so-called *phase averaging*, *dephasing representation* (DR) or *Wigner averaged classical limit*^8,17,18,62,159–164^

$$f_{\text{DR}}(t)=h^{-D}\int dx_{0}\rho_{W}(x_{0})e^{i\Delta S(x_{0},t)/\hbar },$$ (40)

where $x_{0}=(q_{0},p_{0})$ denotes the initial phase-space coordinates of a classical trajectory, $\rho_{W}(x_{0})$ is the Wigner phase-space representation of the initial state |ψ〉, and

$$\Delta S(x_{0},t)=-\int_{0}^{t}\Delta V(x_{t^{\prime }})dt^{\prime }$$ (41)

is the action due to the difference $\Delta V(x_{t^{\prime }}):=V_{2}(x_{t^{\prime }})-V_{1}(x_{t^{\prime }})$ between two potential energy surfaces along the classical trajectory guided by the average Hamiltonian $\bar{H}\equiv (H_{1}+H_{2})/2$. Using the Wigner function $\rho_{W}(x_{0})$ as a sampling weight for the initial conditions *x*_0_, one can rewrite Eq. (40) as a statistical average

$$f_{\text{DR}}(t)={〈e^{i\Delta S(x_{0},t)/\hbar }〉}_{\rho_{W}(x_{0})}.$$ (42)

The most attractive feature of the dephasing representation is that it does not require the calculation of a Hessian along the classical trajectory. Moreover, the number of trajectories required for convergence is independent of the system's dimensionality,^165^ is much lower than the number required in the Herman–Kluk initial value representation, and typically ranges from a hundred to a few thousand.

While the dephasing representation (42) is exact for the displaced harmonic oscillators^8^ and often accurate in chaotic systems,^18^ it breaks down when the Hamiltonians *H*_1_ and *H*_2_ represent the harmonic oscillators with significantly different force constants. To correct this drawback, Zambrano and Ozorio de Almeida^166^ proposed the *dephasing representation with a prefactor* (DRP)

$$f_{\text{DRP}}(t)={〈A_{\text{DRP}}(x_{0},t)e^{i\Delta S(x_{0},t)/\hbar }〉}_{\rho_{W}(x_{0})},$$ (43)

where the prefactor $A(x_{0},t)$ depends on the Hessian of the DR phase $\Delta S(x_{0},t)$ with respect to initial conditions. Consequently, the numerical cost of propagating a single trajectory is higher for DR with a prefactor compared to the original formulation of the DR, but the prefactor correction extends the validity of the approximation.^167^

For the systems with many degrees of freedom, even propagating only a thousand trajectories could be computationally unfeasible. The number of trajectories required to achieve convergence can be reduced by employing smoothing techniques, such as Filinov filtering (cellularization)^145–147^ used for the Herman–Kluk initial value representation. Šulc and Vaníček^168^ and Zambrano *et al.*^167^ proposed a related but somewhat more rigorous approach to the cellularization, which unlike standard Filinov techniques (with two free parameters for position and momentum widths of the cells) has no free parameter and is guaranteed to converge to the original dephasing representation in the limit of the number of trajectories going to infinity. In this method, as in Heller's cellular dynamics, the neighboring trajectories are grouped into cells, and all contributions from the cell are evaluated approximately analytically using the information collected along the central trajectory. This approach yields the *cellular dephasing representation* (CDR)^167,168^

$$f_{\text{CDR}}(t)={〈A_{\text{CDR}}(x_{0},t)e^{i\Delta S(x_{0},t)/\hbar }〉}_{\rho_{\text{IWT}}(x_{0})},$$ (44)

where the prefactor $A_{\text{CDR}}(x_{0},t)$^167^ accounts for the contributions from each cell and the sampling weight for the initial conditions $\rho_{\text{IWT}}(x_{0})$ is given by the inverse Weierstrass transform of the Wigner function $\rho_{W}(x_{0})$.^167^

As the most expensive part of both DR with a prefactor and cellular DR is the calculation of the Hessian of $\Delta S(x_{0},t)$ with respect to the initial conditions, the two methods can be easily combined without increasing the cost per trajectory. The resulting *cellular dephasing representation with a prefactor* (CDRP), which evaluates the correlation function as

$$f_{\text{CDRP}}(t)={〈A_{\text{DRP}}(x_{0},t)A_{\text{CDR}}(x_{0},t)e^{i\Delta S(x_{0},t)/\hbar }〉}_{\rho_{\text{IWT}}(x_{0})},$$ (45)

has a potential to be more accurate and more efficient than the original DR formulation (see Sec. III B 1).

An alternative route for improving the accuracy of the dephasing representation replaces the independent semiclassical trajectories with coupled Gaussian basis functions. This is closely related to the basic idea employed in multiple spawning,^23^ variational Gaussian wavepackets,^169^ coupled coherent states, and multiconfiguration Ehrenfest method.^170^ The time evolved states $|\psi_{j}(t)〉$, *j* = 1, 2 are expanded in the Gaussian basis as

$$|\psi_{j}(t)〉=\sum_{\alpha =1}^{N}c_{j,\alpha }(t)|g_{\alpha }(t)〉,$$ (46)

where $|g_{\alpha }(t)〉$ is the Gaussian wavepacket whose center moves according to the average Hamiltonian $\bar{H}$ and the expansion coefficients $c_{j,\alpha }(t)$ satisfy the time-dependent Schrödinger equation

$$i\hbar S{\dot{c}}_{j}=(H_{j}-i\hbar D)c_{j}.$$ (47)

Here $H_{j}$ is the Hamiltonian matrix, **S** is the overlap matrix, and **D** is the nonadiabatic coupling matrix; their matrix elements in the Gaussian basis are

$$H_{j,\alpha \beta }(t)=〈g_{\alpha }(t)|{\hat{H}}_{j}|g_{\beta }(t)〉,$$ (48)

$$S_{\alpha \beta }(t)=〈g_{\alpha }(t)|g_{\beta }(t)〉,$$ (49)

$$D_{\alpha \beta }(t)=〈g_{\alpha }(t)|{\dot{g}}_{\beta }(t)〉.$$ (50)

In the *Gaussian dephasing representation* (GDR),^171^ the information obtained along the propagated classical trajectories is used to construct the matrices in Eqs. (48)–(50); the time dependence of the expansion coefficients $c_{j,\alpha }(t)$ is then obtained by solving Eq. (47). Finally, the wavepacket correlation function (39) is calculated as^171^

$$f_{\text{GDR}}(t)=c_{2}{\left(t\right)}^{†}S(t)c_{1}(t).$$ (51)

As the size of the Gaussian basis increases and the basis approaches completeness, the result of the Gaussian DR approximation should converge to the exact quantum answer.

Overall, the phase averaging, dephasing representation, and their variants described in this section provide an efficient semiclassical approach for computing wavepacket correlation functions and have found a wide range of applications in molecular spectroscopy.^62,159,161,167,168,171^ Several examples demonstrating both merits and limitations of different extensions of the phase averaging will be provided in Sec. III B 1.

#### 2. Ab initio thawed Gaussian approximation

**FIG. 1.**
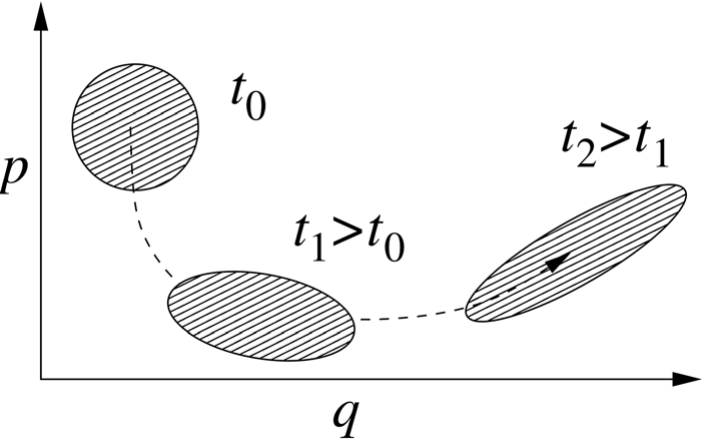
Evolution of a Gaussian wavepacket in phase space within the thawed Gaussian approximation.

One of the simplest, yet efficient, semiclassical approaches for molecular dynamics is provided by the *thawed Gaussian approximation* (TGA) developed by Heller and co-workers.^16,172^ The method is based on the fact that the time evolution of a Gaussian wavepacket in constant, linear, and harmonic potentials does not perturb its functional form. In other words, while it can spread, compress, and rotate in the phase space, a Gaussian remains a Gaussian (Fig. 1).

Thus, within the TGA, the center of a Gaussian wavepacket is guided by a classical trajectory, which accounts for the full anharmonicity of the potential, while the width is propagated using a time-dependent effective potential obtained from a local harmonic approximation of the full potential

$$V_{\text{eff}}(q,t)=V{|}_{q_{t}}+{\left({\text{grad}}_{q}V{|}_{q_{t}}\right)}^{T}\cdot (q-q_{t})+\frac{1}{2}{\left(q-q_{t}\right)}^{T}\cdot {\text{Hess}}_{q}V{|}_{q_{t}}\cdot (q-q_{t}).$$ (52)

Here $V{|}_{q_{t}},\;{\text{grad}}_{q}V{|}_{q_{t}}$, and ${\text{Hess}}_{q}V{|}_{q_{t}}$ are the potential, its gradient, and Hessian evaluated at the center of the Gaussian wavepacket. The evolving wavepacket is assumed in the form^16,172^

$$\psi (q,t)=N_{0}\;\text{exp}\;\left\{-{\left(q-q_{t}\right)}^{T}\cdot A_{t}\cdot (q-q_{t})+\frac{i}{\hbar }\left[{\left(p_{t}\right)}^{T}\cdot (q-q_{t})+\gamma_{t}\right]\right\},$$ (53)

where *N*_0_ is the initial normalization factor, $\left(q_{t},p_{t}\right)$ are the phase-space coordinates of the Gaussian wavepacket's center, *A_t_* is a complex symmetric width matrix, and *γ_t_* is the complex number whose real part gives the phase of the Gaussian wavepacket, while the imaginary part ensures normalization of ψ(q,t) for *t*  >  0. Inserting the ansatz (53) together with the effective potential (52) into the time-dependent Schrödinger equation gives the following equations of motion for the wavepacket's parameters:^16,172^

$${\dot{q}}_{t}={\frac{\partial H}{\partial p}|}_{p_{t}},$$ (54)

$${\dot{p}}_{t}=-{\frac{\partial H}{\partial q}|}_{q_{t}},$$ (55)

$${\dot{A}}_{t}=-2i\hbar A_{t}\cdot m^{-1}\cdot A_{t}+\frac{i}{2\hbar }{\text{Hess}}_{q}V{|}_{q_{t}},$$ (56)

$${\dot{\gamma }}_{t}=L-\hbar^{2}\text{Tr}\left(m^{-1}\cdot A_{t}\right),$$ (57)

where *m* is the diagonal mass matrix, $H=(1/2)p^{T}\cdot m^{-1}\cdot p+V(q)$ is the Hamiltonian, and $L={\dot{p}}_{t}\cdot q_{t}-H$ is the Lagrangian. The numerical integration of the classical equations of motions (54) and (55) is straightforward. The solution of Eqs. (56) and (57) can be simplified^172^ by introducing two auxiliary matrices *Q_t_* and *P_t_* such that

$$A_{t}=\frac{i}{2\hbar }P_{t}\cdot Q_{t}^{-1},$$ (58)

$${\dot{Q}}_{t}=m^{-1}\cdot P_{t}.$$ (59)

In matrix notation, the unique solutions of Eqs. (58) and (59) are given by

$$\left(\begin{array}{c} Q_{t}\\ P_{t} \end{array}\right)=\left(\begin{array}{cc} M_{qq} & M_{qp}\\ M_{pq} & M_{pp} \end{array}\right)\cdot \left(\begin{array}{c} Q_{0}\\ P_{0} \end{array}\right)$$ (60)

with initial conditions $Q_{0}={\text{Id}}_{D}$ and $P_{0}=2i\hbar A_{0}$. Inserting Eqs. (58) and (60) into Eq. (57) and performing the integration yields the explicit solution for *γ_t_* in the form

$$\gamma_{t}=\int_{0}^{t}L\;dt^{\prime }+\frac{i\hbar }{2}\ln\left[\det\left(Q_{t}\cdot Q_{0}^{-1}\right)\right].$$ (61)

Since matrix *Q_t_* is complex, a proper branch of the logarithm has to be taken to make *γ_t_* continuous in time.

Performing calculations with the TGA requires propagating a single classical trajectory, which makes it very useful in implementation with the on-the-fly dynamics; the moderate computational cost allows us to perform molecular dynamics simulations of large systems inaccessible to other methods (see Sec. III B 3). Although the accuracy of a single Gaussian wavepacket description is limited, it can supply the most important information beyond that available in the static calculations employing the global harmonic approximation for the potential^173–176^ or that from purely classical molecular dynamics simulations.

### D. Classical dynamics

Using classical molecular dynamics simulations for investigating the dynamics in the gas- and in the condensed-phase goes back to the late 1950s.^177^ Solving Newton's equations of motions for given initial conditions^178^ and a parametrized energy functions yields coordinates *q*(*t*) and momenta *p*(*t*) from which a multitude of experimentally accessible observables can be determined using statistical mechanics. Compared with a quantum mechanical treatment, nuclear dynamics followed along classical trajectories neglects three essential effects: zero-point energy, tunneling, and coherence. In this context, it is of interest to note that the results for one of the earliest simulations of a reactive process (quasi-classical simulation of the reactive collision of H + H_2_)^179^ have been almost quantitatively confirmed at room temperature by a full quantum treatment some 10 years later.^180^ Hence, even for a system where one would expect quantum effects to be particularly important, quasi-classical trajectory simulations are capable of providing useful insight.

Given this, interest in classical molecular dynamics simulations has shifted more towards realistically describing the intermolecular interactions which has become possible through considerable advances in electronic structure theory. With current computational equipment, it is possible to compute fully dimensional potential energy surfaces for systems such as malonaldehyde (21 degrees of freedom) at the CCSD(T) level with large basis sets and to represent the energies by fitting to a parametrized expression.^181^ Alternatively, fully dimensional potential energy surfaces for smaller systems using reproducing kernels which *exactly* represent the data from electronic structure calculations are possible.^182–185^

On the other hand, such high-accuracy representations are not yet feasible for systems such as proteins for which empirical force fields are being developed. Based on established parametrized forms^186,187^ recent advances include, among others, multipolar^20,21,188–192^ and polarization interactions.^193,194^ Such extensions now allow predictive atomistic simulations for condensed-phase systems.^195^

#### 1. Explicit proton transfer: The MMPT force field

Proton transfer reactions are fundamental in biophysical and biochemical processes. In order to characterize the properties of a shared proton between an acceptor and donor moiety, various experimental methods have been used in the past. One of the most successful approaches is based on optical spectroscopy.^196–200^

Following bond-breaking and bond-formation in simulations based on parametrized, empirical force fields have started with the empirical valence bond (EVB) technique which was particularly relevant to (proton transfer) reactions in solution.^201^ The generalization of EVB to multi-state EVB has played an important role for investigating proton transfer in solution.^202^ The EVB Hamiltonian usually consists of two or more diagonal terms which are force field expressions for all states of interest. The off-diagonal terms are coupling matrix elements which depend parametrically on one (or several) internal coordinate of the system.^203^ This introduces a dependence on the choice of the coordinates which is not always desirable, e.g., if multiple bond rearrangements can occur. Alternatively, a chemical reaction can be followed along time as the progression coordinate, which is the situation encountered in experiments.^204,205^ This is the purpose of adiabatic reactive molecular dynamics (ARMD) which was originally developed for reactions in the condensed phase.^204,206–208^ More recently, ARMD has also been applied to gas-phase systems such as the vibrationally induced photodissociation of sulfuric acid (H_2_SO_4_). Here, the excitation of a higher overtone ($\nu_{9}\leq 4$) of a local OH stretch vibration can lead to photodissociation into water and sulfur-trioxide (H_2_O + SO_3_) on the pico- to nanosecond time scale.^209,210^ However, the ARMD-trajectories were not suitable for final state analysis of the reaction products because they were based on an explicitly time-dependent Hamiltonian which does not conserve total energy during crossing.

Molecular Mechanics with Proton Transfer (MMPT) is a force field-based method which allows bond formation and bond breaking between the transferring hydrogen atom H* and the acceptor or donor atom, respectively.^19^ In this approach, multi-dimensional potential energy surfaces are parametrized from *ab initio* calculations and fit to efficient representations based on Morse potentials. The additional MMPT-energy is written as

$$V_{\text{MMPT}}=V_{0}(R,\rho )+k\cdot \theta^{2},$$ (62)

where *R* is the donor–acceptor distance and *r* is the donor–H* separation. These two variables *R* and *r* are combined into a coordinate *ρ* defined as $\rho =(r-r_{0})/(R-R_{0})\in [0,1]$ with $r_{0}=0.8$ Å and $R_{0}=1.6$ Å. In Eq. (62) the (isotropic) 2 D potential $V_{0}(R,\rho )$ is a superposition of Morse functions. For linear proton transfer, the third coordinate *θ* involves the angle ∠donor−H*−acceptor and is approximated by harmonic function.^19,211^ In the next step, MMPT was extended to non-linear hydrogen bonded motifs as they occur, e.g., in malonaldehyde.^212^ The nonlinear path is described by a displacement d=r· sin θ orthogonal to the proton transfer path and the 3-dimensional potential is $V_{d}(R,\rho ,d)$ which replaces the term $k\cdot \theta^{2}$ in Eq. (62). Adaptation of the MMPT potential to the chemical environment can be achieved through morphing transformations.^213^

#### 2. Atomistic simulations with multipole electrostatics: MTP force fields

Empirical force fields traditionally employ point charge (PC) electrostatics which describe the charge distribution of a molecule using atom-centered partial charges, interacting with one another according to Coulomb's law. In order to efficiently handle the pronounced long range decay of a 1/r interaction, methods such as Ewald summation have been devised to compute long-range electrostatics in periodic systems.^214,215^ Most of the success of atomistic force fields is due to the effectiveness of PC electrostatics in reasonably well approximating the charge distribution around a particular chemical group. However, limitations become apparent in specific systems, e.g., halogens are notoriously challenging for PC force fields as they fail to model the *σ* hole in front of the atom.^216–218^ In general, the lack of anisotropy limits the ability to model specific chemical interactions, such as the need for dummy atoms in certain water models to better reproduce hydrogen-bond interactions.^219,220^ To this end, multipolar (MTP) electrostatics provide a natural and systematic extension to Coulomb interactions, where anisotropy is included as a series expansion with distinct symmetries.

A quantum-mechanical description would be the most rigorous representation of intermolecular interactions. However, practical computational limitations restrict the amount of sampling one would be able to carry out. As an example, for an isolated chromophore in solution, at least several nanoseconds of molecular dynamics simulations are required for converging typical properties such as the radial distribution function *g*(*r*) or its 1d- or even 2d-infrared spectrum. This corresponds to 10^6^ energy evaluations to be carried out with a time step of Δt=1 fs. This is why one resorts to empirical force-fields which allow extensive sampling of configuration space. The validity of the underlying computational model is verified by comparing with reference data from experiments.^221^ Since the relevant dynamics is governed by electrostatic and van der Waals interactions, multipolar and polarizable force fields^193,194^ are necessary for the interpretation of time scales and structural changes at an atomistic level. However, PC-based force fields are not necessarily inferior compared to MTP parametrization depending on the molecule considered and the property studied.^20,21,222^

#### 3. Force field parametrization

**FIG. 2.**
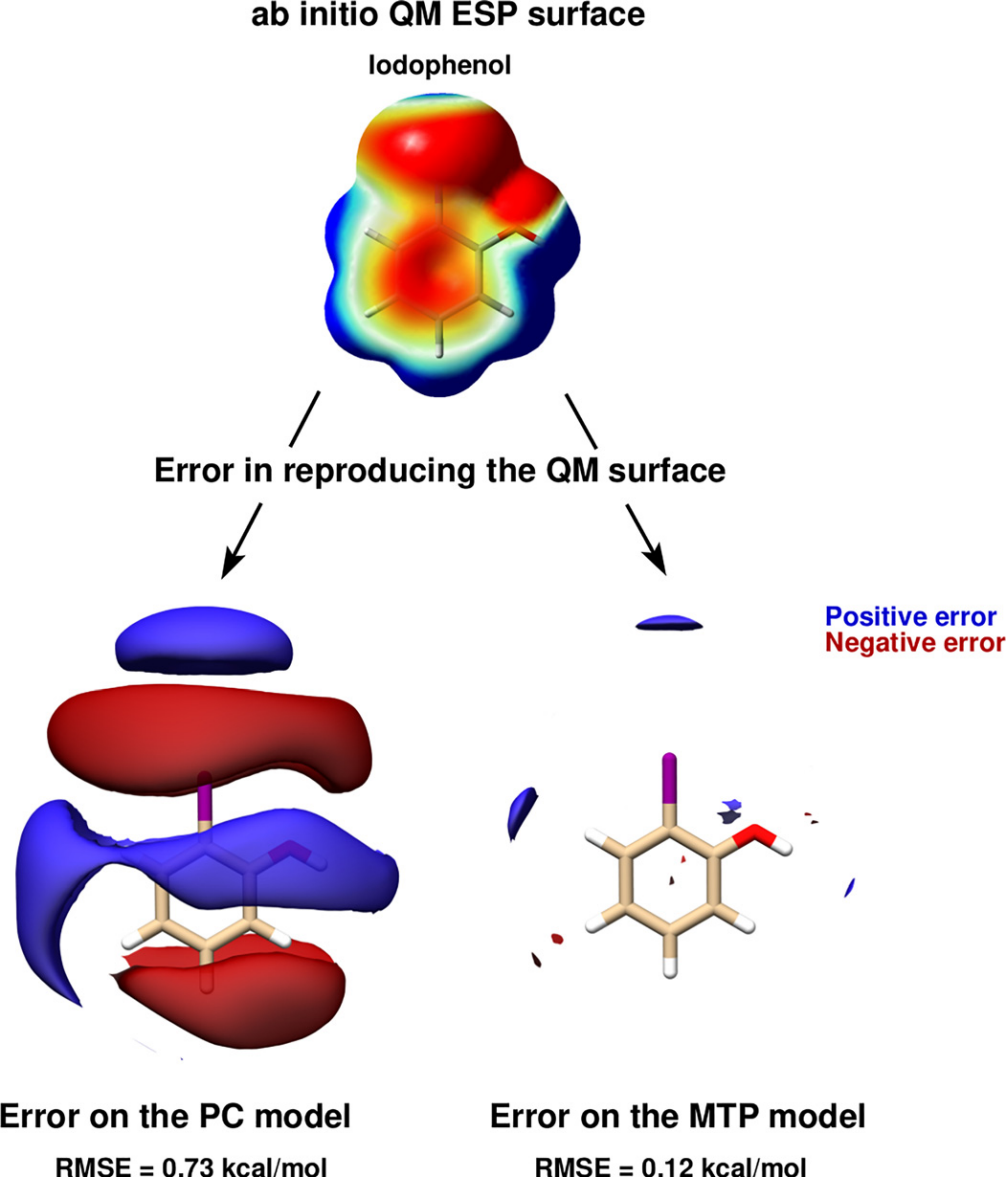
PC vs MTP: Isosurfaces of the difference between *ab initio* and PC and MTP electrostatic potentials for iodophenol (Iodophen-2-ol). Blue and red regions denote the positive and negative errors, respectively. The plots only show points within the first interaction belt.

**FIG. 3.**
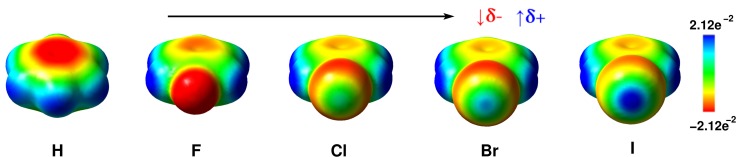
Electrostatic surface potential maps of (form left to right) benzene, fluorobenzene, chlorobenzene, bormobenzene, and iodobenzene at the 10^–3^ e $a^{-3}$ isodensity surface. The color scale of the surface potential ranges from $-2.12e^{-2}$ au (red) to $2.12e^{-2}$ au (blue). The upper black arrow indicates the increase in the sigma-hole strength of the halogens. The red arrow indicates the decrease of the electron rich region $\delta^{-}$ on the sides of the C-halogen bond, and the blue arrow indicates the increase of the electron deficient region $\delta^{+}$ along the C-halogen bond.

**FIG. 4.**
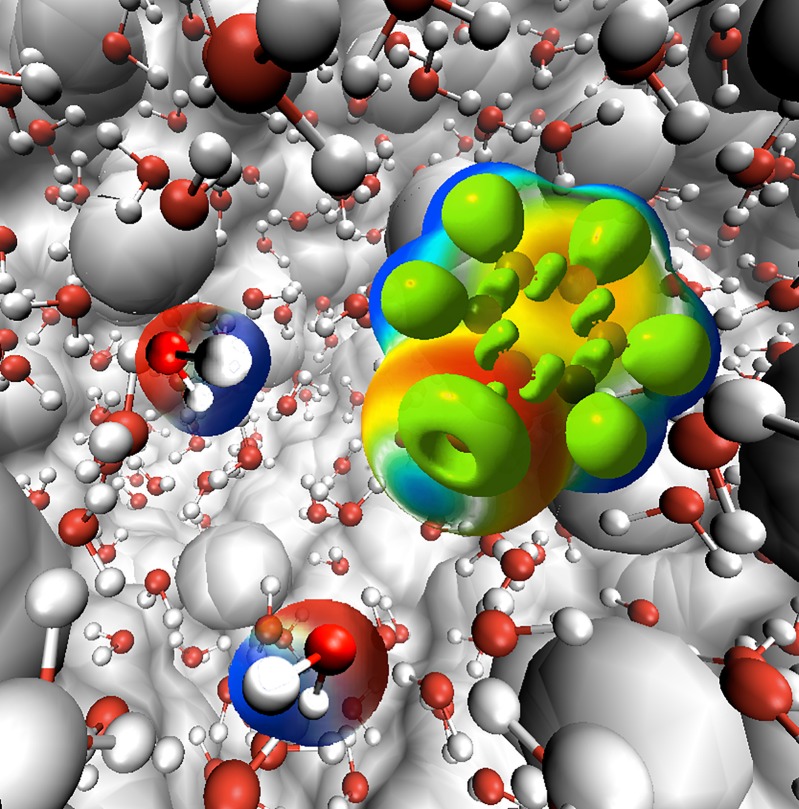
Halobenzene-water dynamics. The figure illustrates the two types of positioning of water molecules around the halogen of halobenzene.

Instead of decomposing the electron density into distributed multipoles,^223^ it is also possible to fit MTP coefficients with respect to the electrostatic potential itself.^224–226^ Expanding the electrostatic potential in terms of the Cartesian coordinates^227^ gives rise to

$$4\pi \varepsilon_{0}\Phi (r)=\frac{q}{R}+\frac{\mu_{\alpha }R_{\alpha }}{R^{3}}+\frac{1}{3}\Theta_{\alpha \beta }\frac{3R_{\alpha }R_{\beta }-R^{2}\delta_{\alpha \beta }}{R^{5}}+\cdots ,$$ (63)

where $1/R\equiv 1/|r-r^{\prime }|$, **r** and $r^{\prime }$ are the locations of the MTP moments, and **r** is an observation point, the Einstein summation convention is applied, and the Kronecker $\delta_{\alpha \beta }$, is 1 only if α=β, and 0 otherwise. Equation (63) shows that the electrostatic potential depends linearly on the MTP coefficients *q*, $\mu_{\alpha }$, etc. Optimizing MTP coefficients to best reproduce the *ab initio* electrostatic potential can thus be done from a linear least-squares fit over a number of discrete points $r^{\left(p\right)}$ around the molecule. In the target function

$$\chi^{2}=\min\sum_{p}\left[\Phi_{\text{ai}}\left(r^{\left(p\right)}\right)-\Phi_{\text{MTP}}\left(r^{\left(p\right)}\right)\right],$$ (64)

the sum runs over a list of discrete points, and $\Phi_{\text{ai}}$ and $\Phi_{\text{MTP}}$ represent the value of the electrostatic potential generated by the *ab initio* and MTP coefficients, respectively. The linearity of the problem allows us to cast $\chi^{2}$ into the form Xb=y, where the matrix **X** represents all geometrical terms (i.e., the *T* tensors^227^) sampled on every grid point, the vector **b** contains all MTP coefficients, and the vector **y** is the collection of *ab initio* electrostatic potential values at every grid point. A comparison between the electrostatic potential and its PC and MTP-representation for iodophenol is shown in Fig. 2. From both the difference density map and the root-mean-square error, it is evident that a PC representation is not capable of correctly describing the electrostatic potential around the molecule. A MTP model is superior by a factor of 5 compared to the PC model and is expected to perform much better in atomistic simulations. This was explicitly shown for an iodinated Tyrosine in insulin complexed to a model for the insulin receptor for which a PC model only leads to one favorable interaction between hormone and receptor, whereas an MTP model establishes 2 additional contacts because the sigma-hole is correctly represented in the MTP model.^195^ A comparison of electrostatic potentials for halogenated benzenes is shown in Fig. 3. In all cases, a MTP representation of the electrostatics together with van der Waals parameters fitted to experimental data yields hydration free energies within 0.1 kcal/mol of the reference values whereas for PC models the difference can be 5 times larger depending on the halogen modification.^20,218^ The choice of the electrostatic model (PC vs. MTP) leads to a different water-ordering around the solute (see Fig. 4) which directly impacts on the quality of the quantity computed from the simulation which is the hydration free energy in this case.

The final parametrization of the nonbonded interactions of a force field involves accurate electrostatic parameters (see above) and optimized van der Waals parameters for condensed-phase simulations. This second step requires explicit molecular dynamics simulations to be run with fixed PC or MTP parameters while adjusting the van der Waals well depths *ϵ* and the ranges *σ* to best reproduce experimentally determined thermodynamic reference data. Often, the pure liquid density, the heat of vaporization, and the hydration free energy of the target molecule are used as the reference to fit to. Starting from, e.g., the CGenFF^228^ force field observables are computed from explicit molecular dynamics simulations. Then, the van der Waals parameters are modified by scalar factors for efficient optimization and the simulations are repeated until a predefined quality of the fit is obtained. Such a procedure has been cast into a versatile computing environment which demonstrated that it is possible to reach an RMSD between experimental observations and computed thermodynamic properties of 0.36 kcal/mol for a range of 20 diverse small molecules can be obtained.^21^

As mentioned above, a MTP representation is not unique and such a fit is usually overdetermined. Hence, the number of MTP components required to best reproduce a reference electrostatic potential can be reduced to obtain a predefined level of accuracy.^229^ Also, symmetries can be exploited to further reduce the number of MTP components.^225^ An alternative to arrive at multipolar-quality force fields is to use the isomorphism between multipoles, atomic orbitals, and their point charge representation. This was exploited in the distributed charge model^230^ which was recently further improved to a minimal distributed charge model^231^ based on off-centered point charges. The minimal distributed charge model is capable of approximating the reference *ab initio* electrostatic potential with an accuracy as good as or better than MTPs without the need for computationally expensive higher order multipoles. For three test cases (imidazole, imidazole cation, and phenylbromide), the best minimal distributed charge model outperforms a multipole expansion truncated after the quadrupole term and is very close to or even better in quality than a multipole expansion truncated after the octupole term. At the same time, a minimal distributed charge model usually uses fewer than two PCs per atom and is therefore computationally more efficient by about one order of magnitude than the corresponding distributed charge model,^230^ while having the same computational advantages over MTPs in molecular dynamics simulations. Remarkably, for imidazole and PhBr, it is even possible to find a minimal distributed charge model with fewer PCs than atoms (i.e., more efficient than a conventional PC representation), which has a quality comparable to a multipole expansion truncated after the quadrupole term.^231^

## III. APPLICATIONS

### A. Quantum dynamics

#### 1. An application of NABDY to the collision of H with H_2_

The theoretical formalism of the NABDY approach, which provides an accurate on-the-fly solution of the electronic and the nuclear time-dependent Schrödinger equations, has already been described above in some details. A small molecular system has been chosen to demonstrate the applicability of the method. To this end, the dynamical problem of collision of H with H_2_ has been found to be a convenient test case to perform NABDY simulations.^115^

The electronic energies and the nonadiabatic coupling vectors have been computed on-the-fly at the DFT/TDDFT level using LDA functional.^232–234^ An energy cutoff of 70 Ry and a cubic box of 20 Bohrs have been employed in all the electronic structure calculations performed with the plane wave code CPMD. The smaller second-order nonadiabatic couplings $D_{ij}^{\gamma }((R))$ were neglected [see Eq. (24)], which, due to the low dimensionality of the problem, do not lead to a considerable norm-conservation problem. Exact wavepacket propagation and TSH calculations have also been performed on the initial wavepacket using the same potential energy surfaces and nonadiabatic couplings obtained with NABDY to compare and validate results. The exact wavepacket propagation has been performed on a fitted one-dimensional surface obtained by the unconstrained NABDY dynamics.

**FIG. 5.**
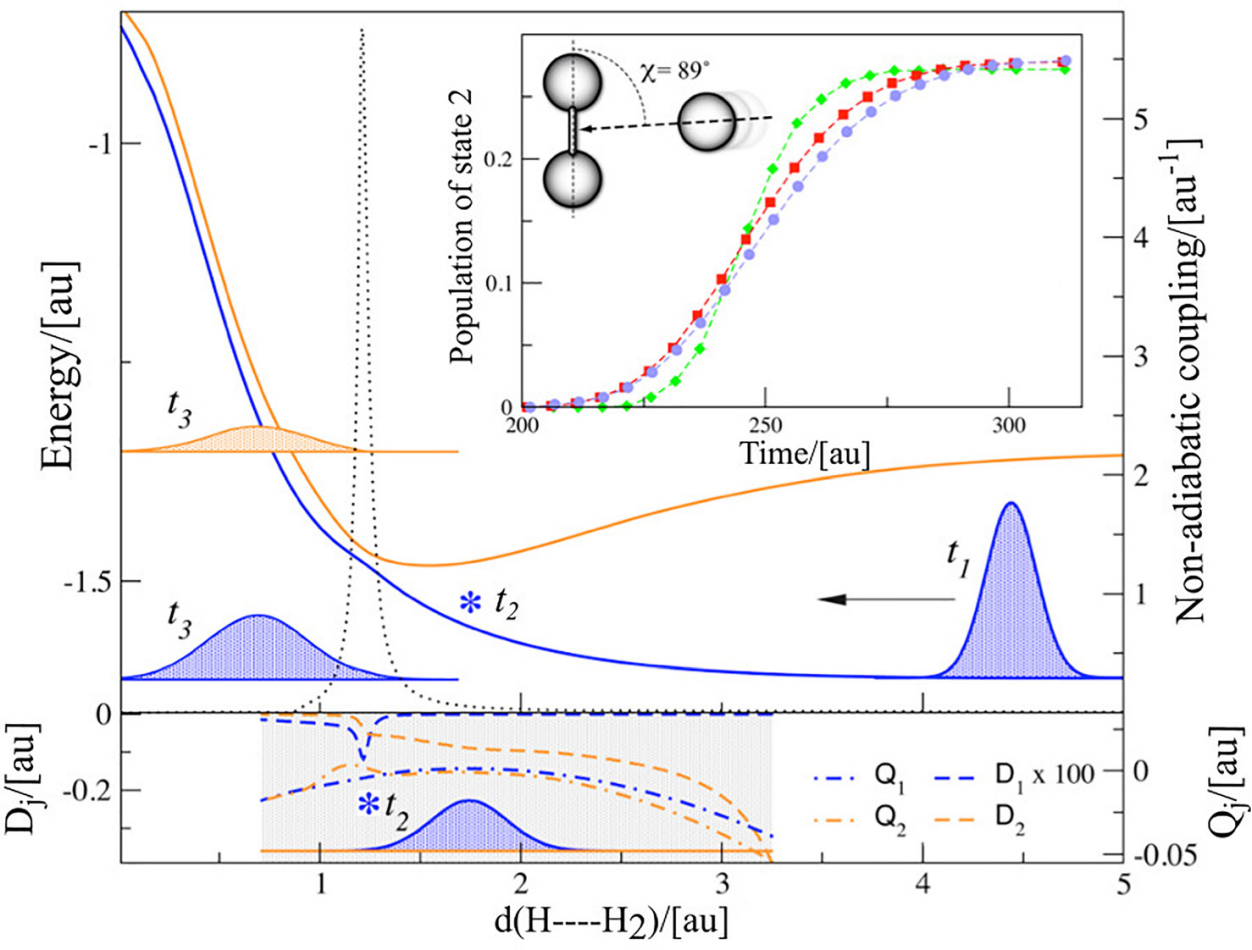
NABDY simulation results for the collision of H with H_2_. Top panel: the H atom approaches the H_2_ molecule with an initial momentum *k* = 75 au along the path which makes an angle χ=89° with the H–H bond axis (see inset). A Gaussian wave packet prepared at $t_{1}=0$ au evolves with time. Shown is the probability density of the nuclear wave packet obtained with 352 trajectories at *t*_1_ and at $t_{3}=300$ au after it crosses the region of strong nonadiabatic coupling. The wavepackets on the different states are indicated with blue (ground state) and orange (excited state) colors. The vertical displacement of the wavepackets at *t*_3_ is arbitrary. The black dotted line represents the nonadiabatic coupling strength. The inset illustrates the time-dependent population of the first excited state obtained with the different dynamics methods. The bottom panel shows the quantum potentials ($Q_{j}$) and the non-adiabatic potentials ($D_{j}$) computed at time *t*_2_ (see the asterisk in the upper panel). At this time, the ground state wavepacket is centered at *d* = 1.75 au.

The system has been prepared with an H atom with an initial momentum *k* = 75 au moving towards the H_2_ molecule along the collision path shown in Fig. 5 (inset). During the course of the dynamics, as the colliding bodies approach each other, they eventually encounter a region of strong nonadiabatic coupling, and electronic population undergoes a partial transfer from ground electronic state to the first excited state as is shown in Fig. 5 (top panel). The amount of transfer has been seen to depend on the momentum of the incident H atom. For *k* = 75 au, NABDY estimates a 27.9% population transfer, whereas the exact propagation gives a 27.8% population in the first excited state. It is worth noting that despite the inherently *ad hoc* stochastic hops and the independent trajectory approximation, the TSH scheme is able to reproduce the excited state populations quite accurately. However, TSH estimates a rate of population transfer slightly higher than the exact one. The agreement on the amount of population transfer for NABDY calculations with the exact result is remarkable. The systematic agreement of the NABDY results with that of the exact one stems from the presence of the adiabatic quantum and the nonadiabatic quantum potentials in the NABDY equations of motion [see Eq. (26)] which are non-existent in TSH. The bottom panel of Fig. 5 shows the quantum and the non-adiabatic quantum potentials calculated as a function of H–H_2_ distance when the ground state wavepacket was centered around *d* = 1.75 au. Overall, it can be concluded that NABDY, being a correlated trajectory method, can capture the additional nuclear quantum effects which is not possible in Tully's surface hopping approach. Interested reader is suggested to consult Ref. 115 for further details.

#### 2. Photoinduced ultrafast intramolecular proton transfer of 4-hydroxyacridine: An application of local control theory

Mixed quantum/classical dynamical methods based on on-the-fly determination of the electronic structure,^235,236^ such as TSH,^237^ are best suited for the application of LCT to photochemical problems of larger systems (such as biomolecules), especially when a specific environment needs to be considered. LCT, when combined with *ab initio* molecular dynamics, carries more appeal as it requires a single forward propagation in time, while conventional optimal control theory typically involves several forward and backward propagations. The TSH/LCT implementation developed in the framework of the NCCR MUST targets typically state-specific electronic transitions.^134,238^ Starting from a system, usually in its ground electronic state, it computes the instantaneous optimal pulse which induces electronic population transfer to the desired state, eventually leading to a trajectory hop from the initial state to the target state.

As an application of this on-the-fly TSH/LCT approach based on an LR-TDDFT framework,^117,238–240^ the photoinduced ultrafast intramolecular proton transfer of 4-hydroxyacridine (4-HA) has been investigated. 4-HA has previously been studied both experimentally and theoretically with static calculations^241,242^ showing that the proton transfer in the ground state is hindered by a prohibitively high potential energy barrier, which is reduced by a large extent in the first excited (S_1_) state. Therefore, to assess the involvement of electronic excited states on the ultrafast dynamics in this system, an unconstrained nonadiabatic *ab initio* molecular dynamics study combined with LCT (such as TSH/LCT) has been performed.

To this end, an isolated 4-HA molecule was placed in a simulation box of dimension 16×16×10 Å. Martins-Troullier-type pseudopotentials^243^ have been employed with a cutoff of 100 Ry for the plane wave basis. The ground and the first three excited electronic states (S_1_, S_2_ and S_3_) have been included in the calculations. To compute the excitation energies and the nuclear forces, the LR-TDDFT equations were solved using the Tamm-Dancoff approximation.^244^ The Perdew-Burke-Ernzerhof (PBE) *xc* functional has been used along with the adiabatic approximation for the corresponding *xc* kernel.^245^ The molecule was equilibrated at 300 K by a Born-Oppenheimer molecular dynamics run in the ground electronic state. Different initial configurations were chosen randomly from the Boltzmann distribution obtained from the ground-state equilibration run. All the calculations have been performed using the CPMD package.^118^

The TSH/LCT calculations were initialized with a 2.4 fs seed pulse of field strength 0.005 au which provides an infinitesimal initial population in the target state. This is essential for an effective LCT dynamics, which otherwise would have a zero field as long as the population of the target state remains strictly zero [see Eq. (35)]. The rest of the LCT dynamics has been carried out with a field strength λ=0.1 au. The field has been calculated at every integration time step for the nuclear equations of motion, which was set to 1 au.

**FIG. 6.**
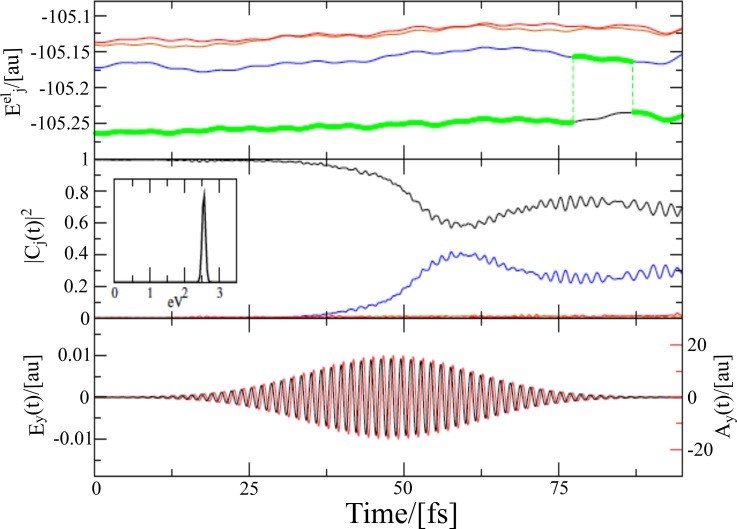
The results of propagating a single trajectory of the TSH/Π-pulse dynamics of 4-HA. Top panel: Potential energies of the ground (black), S_1_ (blue), S_2_ (orange), and S_3_ (red) states as a function of time obtained at DFT/LR-TDDFT level. The green line indicates the driving state, which determines the forces on nuclei during the dynamics. Middle panel: The time-dependent populations of all 4 electronic states for the same trajectory. The inset shows the Fourier transforms of the entire LCT pulse (—). Bottom panel: The LCT pulse in time domain (black line) and the vector potential (red line).

To illustrate the efficiency of the LCT scheme, the results were compared to the case of applying a simple Π pulse (see bottom panel of Fig. 6). To design the Π pulse, we considered a vector potential of the form

$$A(t)=-A_{0}\varepsilon^{\lambda }\;\text{exp}\;\left(-\frac{{\left(t-t_{0}\right)}^{2}}{T^{2}}\right)\text{sin}\;\omega t,$$ (65)

where the frequency *ω* has been chosen to represent the energy gap (2.55 eV) between the ground and the first excited electronic states at the ground-state optimized geometry. The numerical values of the other relevant quantities of Eq. (65) were $A_{0}/c=0.1067$, *t* = 2000 au, and *T* = 800 au, respectively. The results from the propagation of a single trajectory, using the same initial conditions as the TSH/LCT propagation, are depicted in Fig. 6. It shows a smooth transfer of population from the ground to the first excited state for the first 50 fs with an accumulation of 42% population. However, beyond this point, the dynamics exhibits merely oscillatory, back and forth, incomplete transfer of population between the lowest two electronic states (middle panel of Fig. 6). It can also be seen (top panel of Fig. 6) that the trajectory undergoes an actual hop to the first excited state at t∼77 fs but stays there only for a short period of time. Overall, it can be concluded that with this rather naive and weak Π pulse, it is not possible to efficiently promote the population of the ground state to the first excited state selectively.

**FIG. 7.**
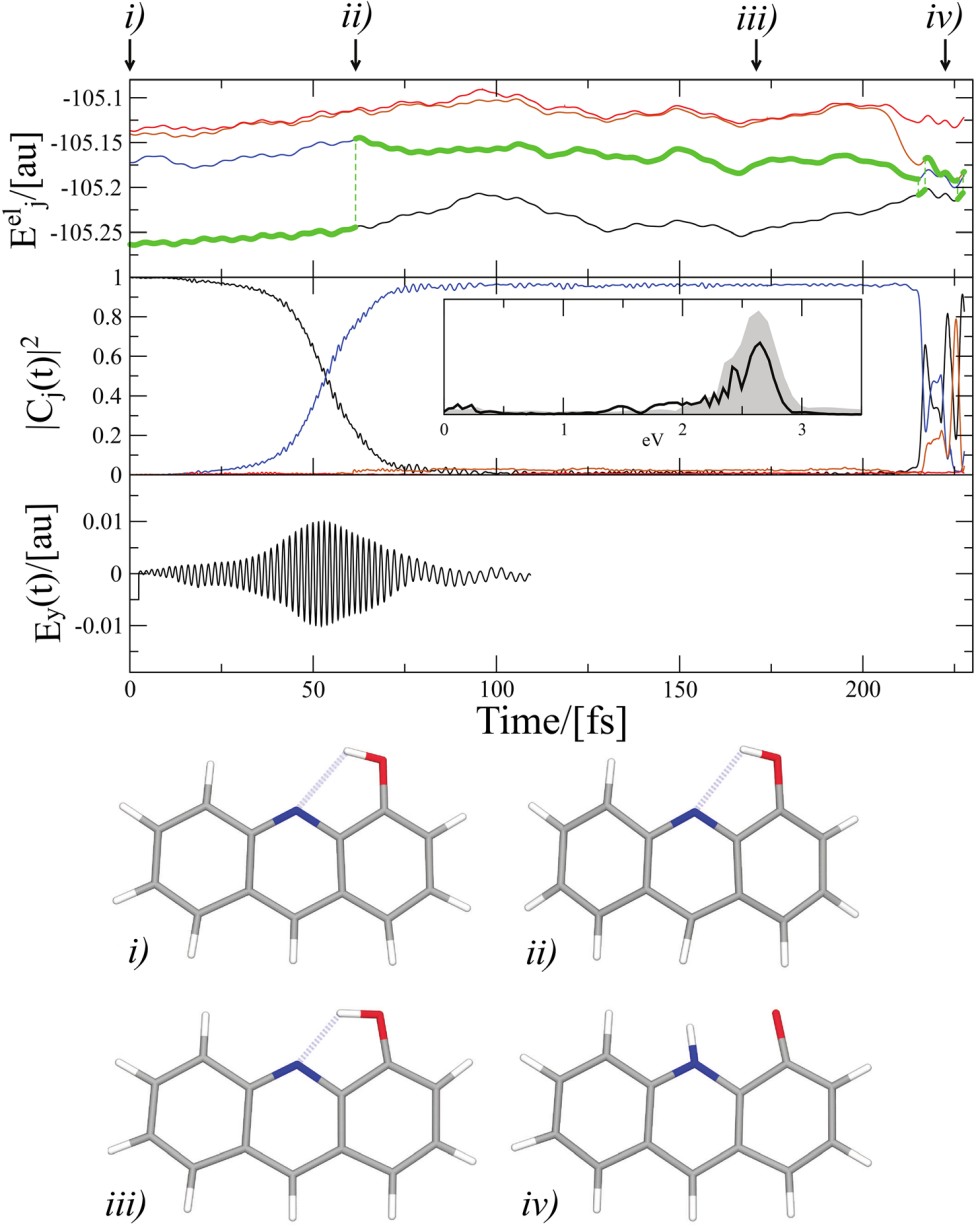
A representative trajectory of the TSH/LCT dynamics of 4-HA. Top panel: Potential energies of the ground (black), S_1_ (blue), S_2_ (orange), and S_3_ (red) states as a function of time obtained at DFT/LR-TDDFT level. The green line indicates the driving state, which determines the forces on nuclei during the dynamics. Middle panel: The time-dependent populations of all 4 electronic states for the same trajectory. The inset shows the Fourier transforms of the entire LCT pulse (—) and the same for the first part of the pulse until the first trajectory hop occurs (light grey area). Bottom panel: The LCT pulse in time domain. Panels (i)–(iv) report 4 representative 4-HA structures sampled along the trajectory (labels correspond to times indicated in the top panel).

At contrast, as it can clearly be seen from Fig. 7, the LCT pulse starts gaining amplitude since the very early stage of simulation and attains a maximum amplitude at ∼50 fs while the corresponding trajectory undergoes a hop to the S_1_ state at ∼60 fs. Consequently, a smooth and almost complete electronic population transfer is achieved within the first 75 fs. The frequency spectrum of the LCT pulse (obtained by Fourier transform) is centered around the vertical energy gap between the ground and the S_1_ state (2.6 – 2.65 eV). Some low-intensity additional peaks appear below 2 eV which mainly stem from the vibrational relaxation within the S_1_ state. The bottom most panel of Fig. 7 depicts 4 representative structures of 4-HA which correspond to 4 important instants of time (shown in the top panel of Fig. 7) during the course of the dynamics. The system starts evolving in time in the ground electronic state with the proton attached to the oxygen atom. At about ∼60 fs, it undergoes a trajectory hop to the S_1_ state of $\pi \pi^{*}$ character [Fig. 7(ii)] which induces a transfer of electron density from the donor (oxygen) atom to the acceptor (nitrogen) atom. Consequently, the N–H distance shortens and the O–H distance increases with time [Figs. 7(iii) and 8] which finally leads to a complete proton transfer, occurring shortly after 200 fs [Figs. 7(iv) and 8]. It is worth noting that only a small amount of population has been seen to accumulate in the other two excited states during the dynamics. Moreover, the occasional hops to these states are always followed by a subsequent quick deactivation to the ground state. Further details about this study can be found in Ref. 246.

**FIG. 8.**
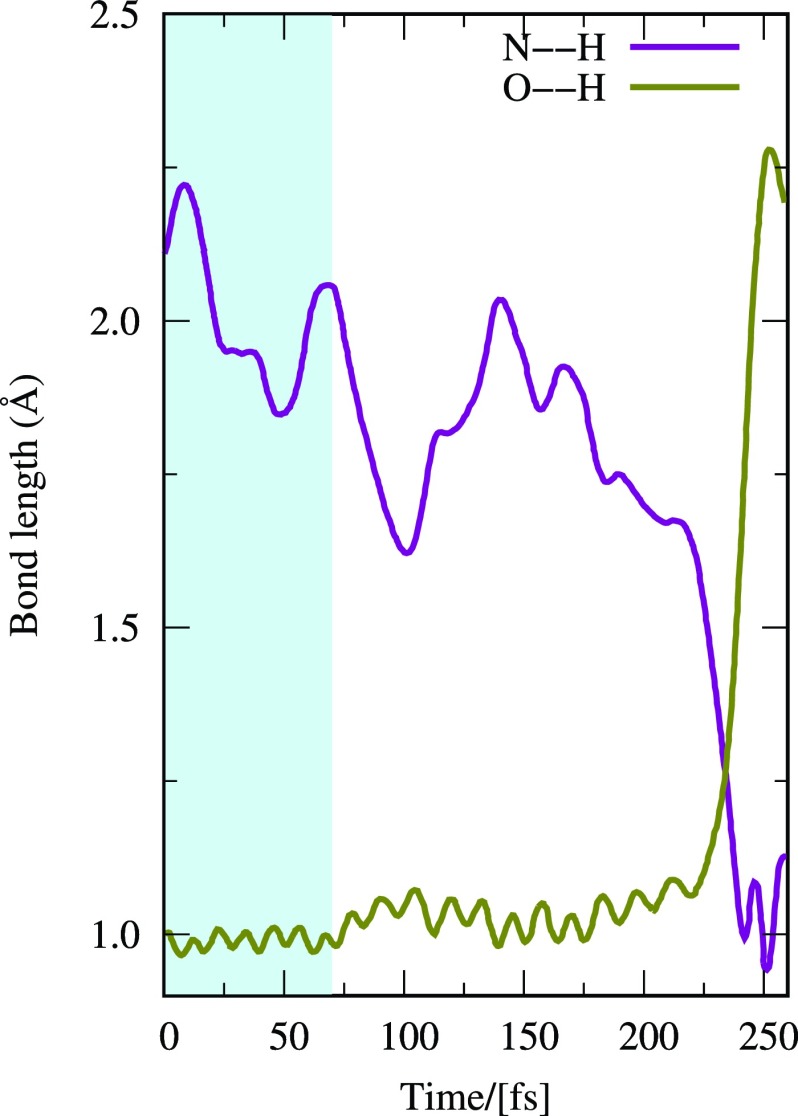
Time evolution of the O–H and the N–H distances of 4-HA along the TSH/LCT dynamics. The cyan area represents the duration in which the molecule is in its electronic ground state.

### B. Semiclassical dynamics

#### 1. Time-resolved stimulated emission spectra of pyrazine

The present section illustrates the performance of several variants of the dephasing representation (Sec. II C 1) in calculating the time-resolved stimulated emission spectra of pyrazine. The employed model^247^ considers the transitions between *S*_0_ and *S*_1_ electronic states and takes into account four normal modes of pyrazine. The nonadiabatic couplings between *S*_1_ and *S*_2_ states are neglected since those do not play a significant role for an $S_{0}\to S_{1}$ excitation.

Assuming the validity of the zero-temperature, electric dipole, and Condon approximations (see Sec. II A 1), assuming the two pulses to be ultrashort and well separated (see Sec. II A 3), and using the time-dependent perturbation theory (see Sec. II A 2), the time-resolved stimulated emission spectrum at the frequency *ω* can be calculated as the Fourier transform

$$\sigma (\omega ,\tau )\propto \text{Re}\int_{0}^{\infty }f(t,\tau )e^{i\omega t}dt$$ (66)

of the wavepacket correlation function^59,86,168^

$$f(t,\tau )=〈\psi_{1}(t,\tau )|\psi_{0}(t,\tau )〉,$$ (67)

where *τ* is the time delay between pump and probe pulses, *t* is the time elapsed after the probe pulse, and

$$|\psi_{j}(t,\tau )〉=e^{-i{\hat{H}}_{j}t/\hbar }e^{-i{\hat{H}}_{1}\tau /\hbar }|\psi 〉$$ (68)

represents the state |ψ〉 (initially the vibrational ground state of the ground state Hamiltonian ${\hat{H}}_{0}$) evolved first for the time delay *τ* with the excited state Hamiltonian ${\hat{H}}_{1}$ and subsequently for time *t* with either the ground or excited state Hamiltonian (*j* = 0, 1; note that we now number the electronic states and corresponding Hamiltonians starting from 0 instead of 1, to agree with the convention of numbering electronic singlet states *S*_0_ and *S*_1_).

The dephasing representation and its variants described in Sec. II C 1 can be easily applied to Eq. (67). The only minor modification consists in replacing the action difference in Eq. (42) with its generalized version^167^

$$\Delta S(z_{0},\tau ,t)=\int_{\tau }^{\tau +t}\Delta V(z_{t^{\prime }})dt^{\prime },$$ (69)

where the classical trajectory $z_{t^{\prime }}$ follows the excited state Hamiltonian *H*_1_ for $t^{\prime }\in [0,\tau ]$ and the average Hamiltonian $\bar{H}$ for $t^{\prime }>\tau$.

**FIG. 9.**
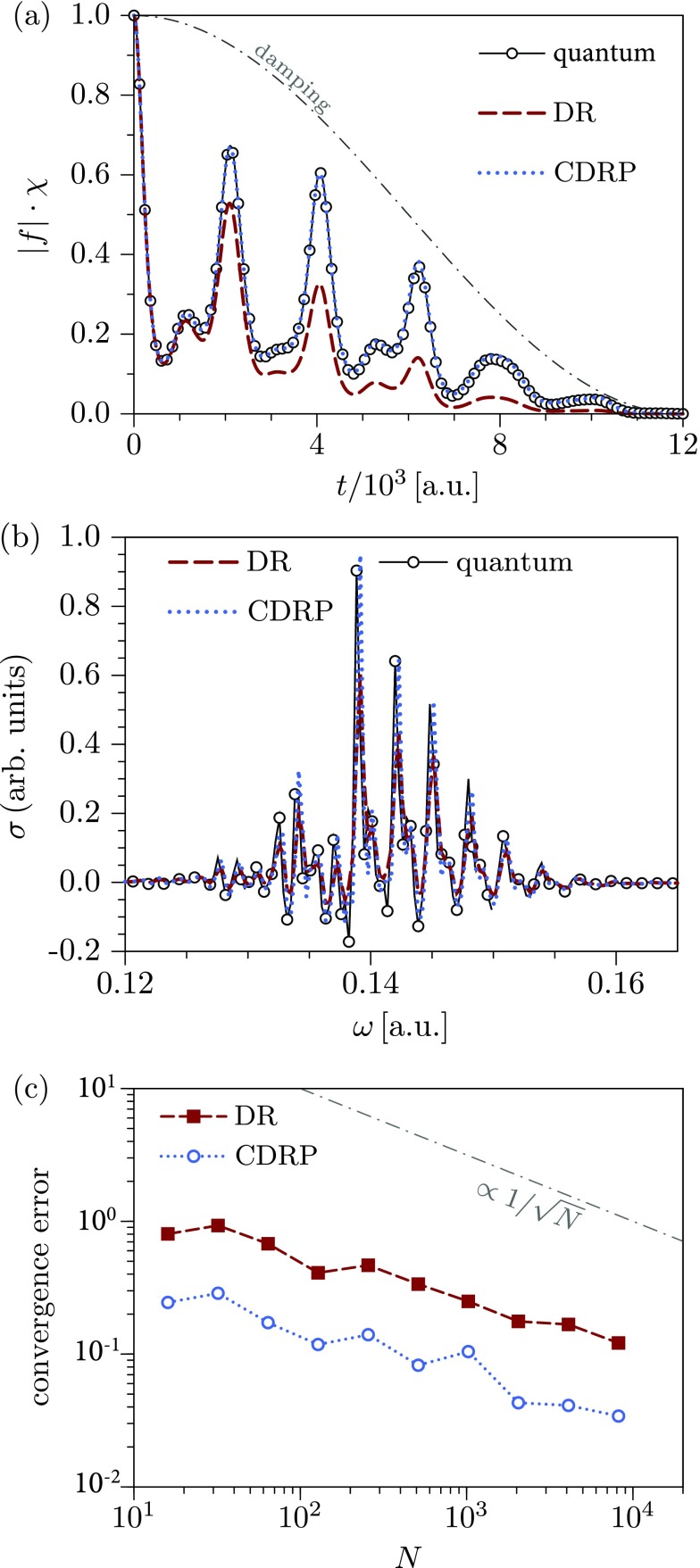
Time-resolved stimulated emission (TRSE) in the pyrazine $S_{0}/S_{1}$ model^247^ for the time delay $\tau =2\times 10^{3}$ a.u.≈48 fs. Comparison of the results of the dephasing representation (DR) and cellular dephasing representation with a prefactor (CDRP) with the exact quantum results. (b) TRSE spectrum. (c) Convergence (measured by the relative *L*^2^ norm error) of the damped correlation function as a function of the number of trajectories *N*. Reprinted with permission from J. Vaníček, Chimia **71**, 283 (2017). Copyright 2017 Swiss Chemical Society.

Figure 9 compares the time-correlation functions and spectra calculated using the DR and cellular DR with a prefactor with the corresponding exact quantum-mechanical results. The cellular DR with a prefactor agrees remarkably well with the quantum calculation [see panels (a) and (b)] and requires fewer trajectories for convergence than the original DR [see the convergence plot in panel (c)]. However, the latter property is not universal—in strongly chaotic systems, such as the quartic oscillator, a few chaotic trajectories with very large prefactors may require an enormous number of well-behaved trajectories to compensate for this, whereas the original DR approach avoids this issue since it contains no potentially problematic prefactors.^167^

**FIG. 10.**
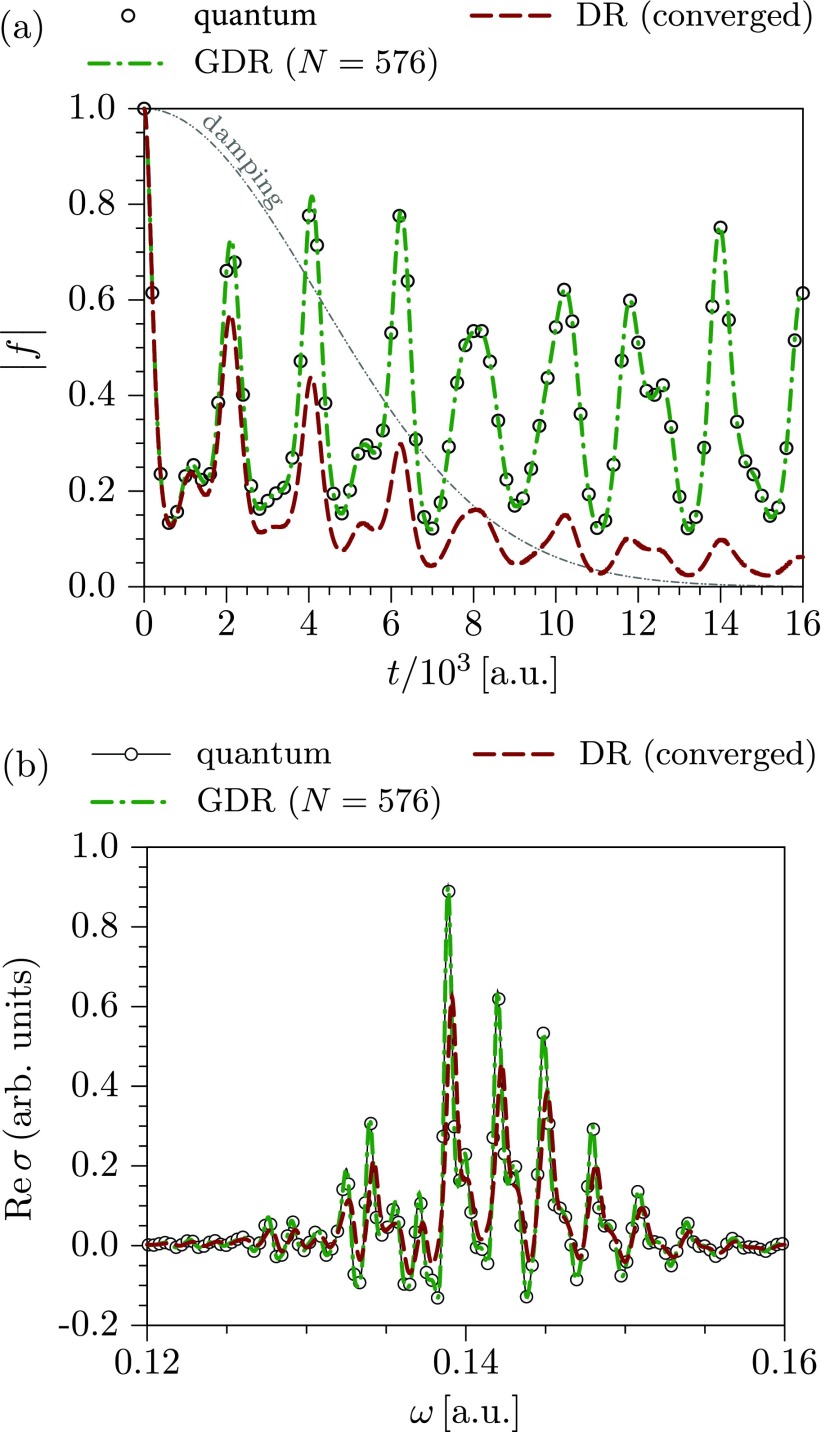
Time-resolved stimulated emission (TRSE) in the pyrazine $S_{0}/S_{1}$ model^247^ for the time delay $\tau =2\times 10^{3}$ a.u. ≈48 fs. Comparison of the results of the dephasing representation (DR) and Gaussian DR (GDR) with the exact quantum results. (a) Time correlation function. (b) TRSE spectrum obtained as a Fourier transform of the correlation function multiplied by a damping function displayed in panel (a) by a gray dashed-double-dotted line. Reprinted with permission from J. Vaníček, Chimia **71**, 283 (2017). Copyright 2017 Swiss Chemical Society.

The accuracy of the Gaussian dephasing representation is demonstrated on the same pump-probe system in Fig. 10. The correlation function and spectrum computed with the Gaussian dephasing representation and using 576 basis functions are virtually indistinguishable from the exact quantum results, unlike the fully converged DR calculation, which contains a residual semiclassical error. We also note that, while the original DR does not capture the absolute magnitudes of all peaks in the spectrum correctly, the positions and relative intensities are described rather well with the DR [see Figs. 9(b) and 10(b)]. Thus, even the original DR (or phase averaging) provides a computationally efficient tool for a qualitative prediction of molecular spectra since no Hessians are required.

#### 2. Absorption and photoelectron spectra of ammonia

In this section, we illustrate the performance of the on-the-fly *ab initio* TGA method (Sec. II C 2) in describing the spectra of floppy molecules, i.e., molecules in which one would expect a local harmonic approximation to break down due to large amplitude, anharmonic motions. Ammonia (NH_3_) is used as a representative example of a floppy molecule, which, due to its small size, allows for comparison of different and rather accurate levels of *ab initio* theory, permitting to separate the errors due to electronic structure evaluation from those due to the dynamical approximation.

Within the Born-Oppenheimer, zero-temperature, electric dipole, and Condon approximations, and using the time-dependent perturbation theory (Sec. II A), the absorption spectrum as a function of the incident light frequency *ω* is obtained as the Fourier transform

$$\sigma (\omega )=\frac{2\pi }{3\hbar c}\mu_{01}^{2}\omega \int dt\;e^{i(\omega +E_{0,0}/\hbar )t}C(t).$$ (70)

Here, *μ*_01_ is the transition dipole moment between the ground and excited electronic states and

$$C(t)=〈\psi_{0,0}|e^{-i{\hat{H}}_{1}t/\hbar }|\psi_{0,0}〉$$ (71)

is the autocorrelation function of the initial ground vibrational state $|\psi_{0,0}〉$ of the ground electronic state with energy $E_{0,0}$, which evolves with excited-state Hamiltonian ${\hat{H}}_{1}$ after the excitation.

**FIG. 11.**
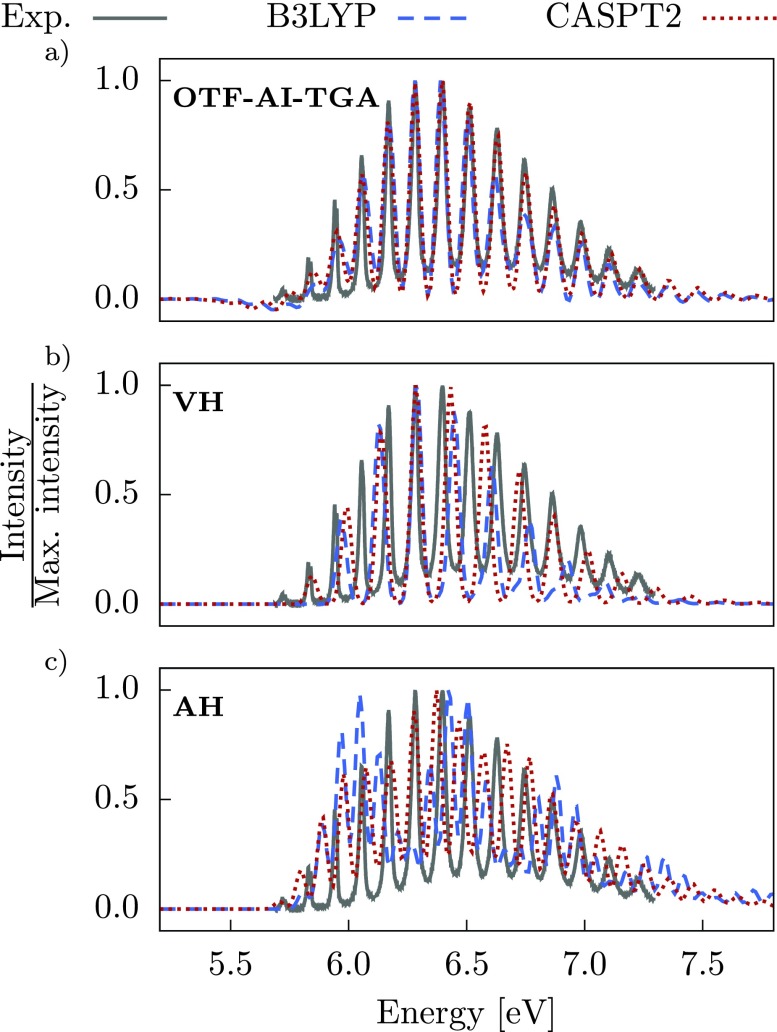
Absorption spectrum of NH_3_: Comparison of the experimental spectrum recorded at the temperature of 175 K with the spectra computed with the on-the-fly *ab initio* thawed Gaussian approximation (OTF-AI TGA), vertical harmonic (VH), and adiabatic harmonic (AH) models within the B3LYP and CASPT2 *ab initio* methods. All spectra are rescaled so that the highest spectral peak in each spectrum is of unit intensity. Reprinted with permission from Wehrle *et al.*, J. Phys. Chem. A **119**, 5685 (2015). Copyright 2015 American Chemical Society.

The experimental ${\tilde{A}}^{1}A_{2}^{\prime \prime }\leftarrow {\tilde{X}}^{1}A_{1}^{\prime }\;(S_{1}\leftarrow S_{0})$ absorption spectrum of ammonia contains a single long progression due to the activation of the umbrella motion of NH_3_ (Fig. 11). The electronic transition under consideration is accompanied by a substantial change of the nuclear configuration from non-planar (${\tilde{X}}^{1}A_{1}^{\prime }$ state) to planar (${\tilde{A}}^{1}A_{2}^{\prime \prime }$ state), which induces a large-amplitude nuclear motion exploring anharmonic regions of the excited potential energy surface.

Figure 11 compares the experimental spectrum with spectra calculated with the on-the-fly *ab initio* TGA approach using CASPT2 and B3LYP levels of theory.^176^ The local harmonic approximation employed in the on-the-fly *ab initio* TGA captures partially the anharmonicity of the potential energy surface of the excited electronic state, resulting in an excellent peak spacing in the corresponding spectrum and the relative intensity distribution. We also note that the employed level of *ab initio* theory mainly affects the intensities of the peaks without modifying the spacing. In addition, Fig. 11 illustrates the performance of the common approach based on the global harmonic approximation for the excited state potential, which is obtained using the *ab initio* data (potential, forces, and Hessian) calculated either at the ground (“vertical harmonic” model) or excited (“adiabatic harmonic” model) state equilibrium geometries.^248^ In the adiabatic harmonic model, the stretching modes are overly excited due to their coupling to the bending mode, which results in unphysical progressions. Furthermore, small changes in the equilibrium geometries caused by employing different levels of the *ab initio* theory have a drastic impact on the spectrum. The vertical harmonic model suffers much less from these two problems and, in addition, it obviously provides a better description of the Franck–Condon region important for spectra calculations. Still, it is clear that neither of the two harmonic models can reproduce the anharmonic peak spacing, while the on-the-fly *ab initio* TGA approach provides a good quantitative description of the absorption spectrum of NH_3_.

A more strict test of the robustness of the on-the-fly *ab initio* TGA was provided by the simulation of the photoelectron spectrum of NH_3_.^176^ This better-resolved spectrum depends on much longer dynamics than does the absorption spectrum and, as a result, the photoelectron spectrum is much more affected by nonlinearity. Indeed, as shown in Ref. 176, the global harmonic approaches break down even more than in the case of absorption spectrum, the vertical harmonic model yielding again too large level spacing and adiabatic harmonic model exhibiting unphysical progressions. Surprisingly, the on-the-fly *ab initio* TGA result agrees with the experimental spectrum reasonably well: the peak positions are almost indistinguishable, whereas the discrepancies in the intensities reflect the deteriorating quality of the local harmonic approximation. Overall, the on-the-fly *ab initio* TGA approach provides a powerful tool for the simulation of the electronic spectra even for floppy systems as long as the contributing dynamics is rather short.

#### 3. Emission spectra of oligothiophenes

Polythiophenes and their functional derivatives demonstrate remarkable conductivity with excellent thermo- and chemo-stability making them very promising for applications in organic electronics. Thus, understanding structural and dynamical properties of such systems is important for the design of new materials. Due to the large size of oligothiophenes, the molecular dynamics simulations using quantum mechanical methods are unfeasible and one is forced to find a compromise between accuracy and computational efficiency.

**FIG. 12.**
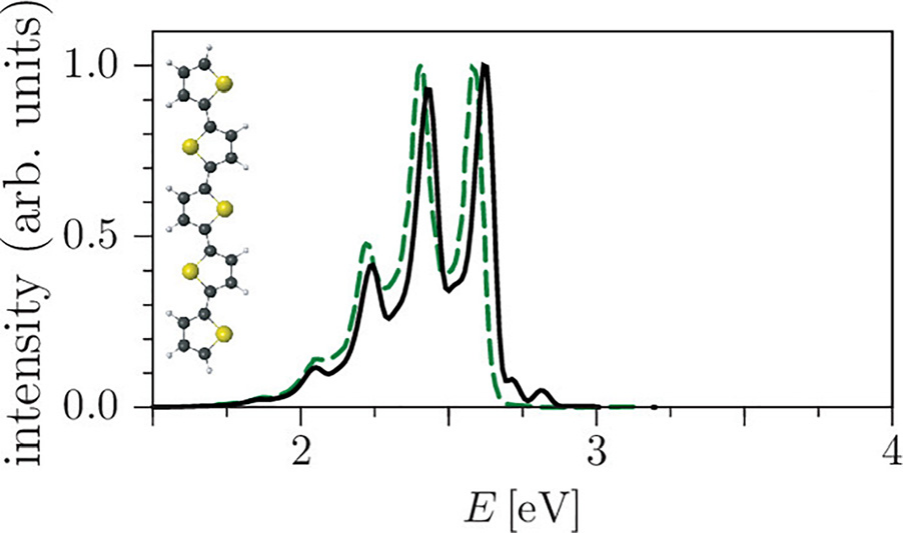
Comparison of the experimental emission spectrum of pentathiophene (dashed green line) with the full-dimensional on-the-fly *ab initio* thawed Gaussian approximation calculation using all 105 normal modes (solid black line). Adapted with permission from J. Chem. Phys. **140**, 244114 (2014). Copyright 2014 AIP Publishing LLC.

The utility of the on-the-fly *ab initio* TGA approach (coupled with DFT and time-dependent DFT electronic structure methods) for predicting the electronic emission spectra of the oligothiophenes (T*n*, where n=2, 3, 4, 5 is the number of thiophene units) has been validated by Wehrle *et al.*^175^ Figure 12 compares the experimental and calculated emission spectra of pentathiophene; both the overall shape of the spectrum and peak intensities are in an excellent agreement. The calculated spectrum is slightly shifted compared to the one experimentally measured, which is most likely due to insufficiently accurate electronic structure methods. Nevertheless, the observed agreement is remarkable considering that the pentathiophene has 105 degrees of freedom, which is currently the largest chemical system treated with the on-the-fly *ab initio* semiclassical dynamics.

To better understand the underlying dynamics, Wehrle *et al.*^175^ proposed a systematic way to analyze the influence of different normal modes on the vibrational structure of the emission spectrum. The method uses components of the stability matrix calculated along the trajectory to partition all normal modes into approximately uncoupled groups and then selects the most important modes by considering the maximum displacements relative to the associated Gaussian width parameters. As a result, this method allows an automatic and natural construction of reduced dimensionality models of complex polyatomic systems.

**FIG. 13.**
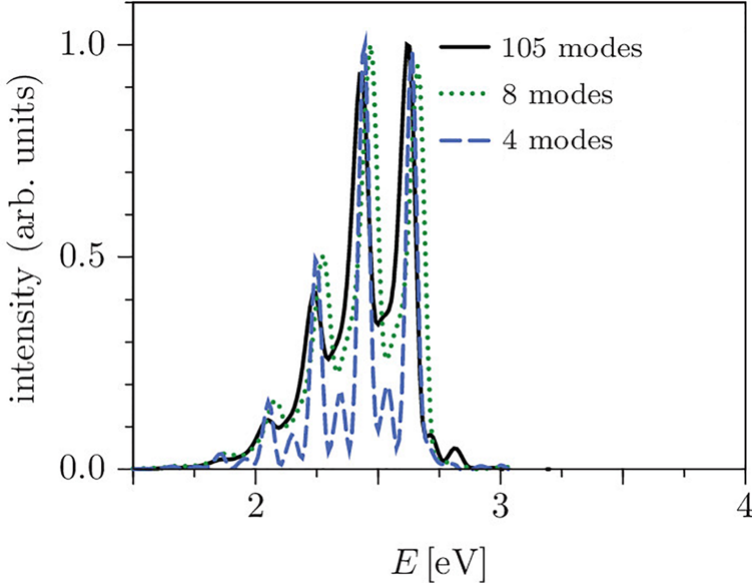
Emission spectrum of pentathiophene: comparison of the spectrum obtained from the full-dimensional on-the-fly *ab initio* thawed Gaussian approximation with reduced dimensionality models generated automatically from a single thawed Gaussian trajectory. Adapted with permission from J. Chem. Phys. **140**, 244114 (2014). Copyright 2014 AIP Publishing LLC.

Figure 13 illustrates the usefulness of this approach on the emission spectrum of pentathiophene, by comparing the full, 105-dimensional result with the results of two, automatically generated models of reduced dimensionality. It is clear from the figure that performing the dynamical simulation with only four active modes, corresponding to an inter-ring stretch and ring-squeeze, yields a good qualitative description of the positions and intensities of all peaks in the 105-dimensional calculation. Moreover, including only four additional modes, attributed to the chain and C-H bond deformations, captures most of the peak broadening and brings the calculated spectrum to an almost perfect agreement with the result of the full-dimensional simulation, which, as we have seen in Fig. 12, describes fully the experimental emission spectrum. Thus, the on-the-fly *ab initio* TGA, combined with the proposed scheme to estimate the importance of normal modes in the dynamics, provides a powerful tool for calculation and analysis of electronic spectra in large molecular systems. Moreover, a single TGA trajectory could be used to factorize the original system into several independent, lower dimensional systems, which can be treated by more accurate or even exact quantum dynamics methods.

### C. Classical dynamics

#### 1. Computational infrared spectroscopy for H-bonded systems

Infrared spectroscopy is a powerful method to characterize the dynamics of molecules in the gas- and condensed phase. For H-bonded systems, the hydrogen-stretch is a particularly sensitive degree of freedom. The energetics and dynamics of proton and hydrogen transfer is of fundamental importance in biology and chemistry.^249–251^ Such processes are primarily governed by the height of the barrier for proton/hydrogen transfer which is, however, difficult to determine reliably through direct experimentation. Possibilities include high resolution spectroscopy where the splitting of spectral lines can provide information about the barrier height^252^ or nuclear magnetic resonance (NMR) experiments.^253,254^ On the other hand, kinetic isotope effects or shift of vibrational bands in the infrared alone cannot be used directly to determine the energetics for proton transfer.

Large-amplitude motion (including proton- or hydrogen-transfer) along the X–H* stretching coordinate in systems containing $X-H^{*}\cdots \;Y$ motifs—where X and Y are the donor and acceptor atoms, respectively, and H* is the transferring hydrogen—can lead to characteristically broadened features in vibrational spectra.^255–257^ This broadening reflects strong coupling between the X–H stretch and other framework modes of the environment and structural heterogeneity.^199^ The broadening even persists down to low temperatures and cooling the species does not lead to sharper bands.^258^

**FIG. 14.**
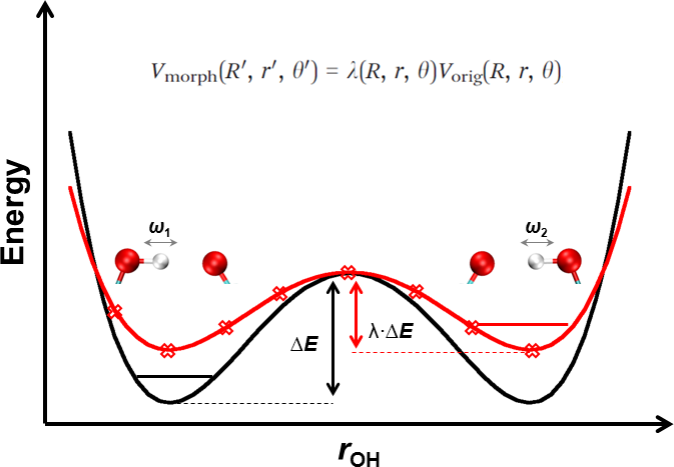
The empirical correlation between morphed potential energy surface and bond stretching frequencies.

As an example, the infrared and near-infrared spectra of acetylacetone^200^ were investigated computationally and through experiments. The fundamental OH-stretching bands were red-shifted relative to those of usual OH stretching transitions. Using a suitably morphed MMPT force field, the computed spectra from atomistic simulations of acetylacetone in the gas phase can be matched with the experimentally determined spectra. The OH-stretching (or proton transfer) band was found to be broad and weak. Furthermore, the wavenumber of this band sensitively depends on the barrier height for proton transfer (see Fig. 14). From comparing computed and experimentally measured infrared spectra, a barrier height of around 2.5 kcal/mol was inferred, which favorably compares with 3.2 kcal/mol obtained from CCSD(T)/cc-pVTZ calculations.^200^

**FIG. 15.**
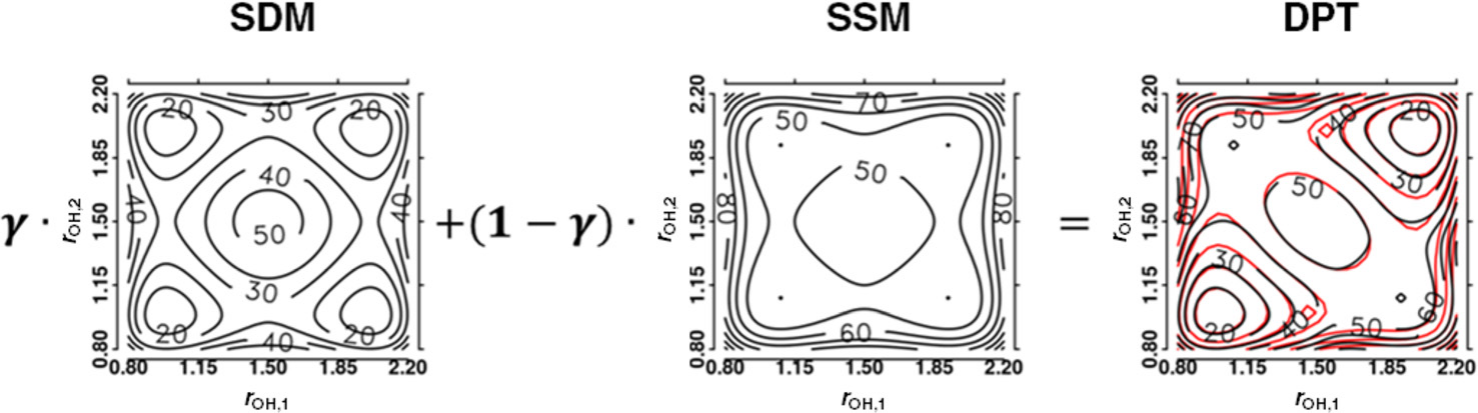
Mixed two-dimensional potential energy surfaces for double proton transfer (DPT) in formic acid dimer. The reference data from MP2 calculations are in red and the empirical potential in black. The right hand panel illustrates that the empirical surface is of very high quality.

MMPT with suitably morphed potential energy surfaces was also employed to analyze the gas-phase infrared spectra of formic acid dimer (FAD)^198^ and of protonated oxalate.^259^ For FAD, a combination of a symmetric double (SDM) and single minimum (SSM) surface yields a realistic description of the double proton transfer potential energy surface (see Fig. 15).^198^ Conversely, for protonated oxalate, the two resonance forms of the molecule can be parametrized such that the change in bonding character of the CO-subunit (from single. to double-bonded) upon proton transfer is incorporated into the energy function.^259^ For both systems (FAD and oxalate), the comparison with experimentally determined infrared spectra in the region of the proton transfer band yields accurate barrier heights of 5–7 kcal/mol and 4.2 kcal/mol, respectively. Hence, estimation of the proton transfer barrier height from a combined computational/infrared spectroscopy approach is likely to be a generic way forward for better characterizing this important quantity for a range of donor-acceptor pairs.

#### 2. Multipolar force fields applications in the condensed phase

In the following, a number of applications of multipolar force fields to spectroscopic and dynamical properties in the condensed phase are described.

##### a. CO in Myoglobin

The use of MTP electrostatics has been of particular relevance in spectroscopic applications, specifically when quantitative comparisons with experiments and their interpretation were of interest. One of the noticeable examples is the infrared spectrum of photodissociated carbon monoxide (CO) in myoglobin. The strong [43 MV/cm (Ref. 260)] inhomogeneous electric field in the heme pocket leads to characteristic shifting and splitting of the spectral lines due to the Stark effect. Several attempts were made^261–263^ to correctly interpret the experimentally known infrared spectrum^264^ using computational methods. Although some of them were capable of correctly modelling the width of the experimentally determined spectrum, they usually were unable to find the characteristic splitting of the CO spectrum (i.e., ≈10 cm^–^^1^). The first successful attempt used a fluctuating point-charge model based on an earlier three-point model for CO.^188,265^ This was later generalized to a rigorous fluctuating MTP model which reproduced most features of the spectrum known from experiments.^189^ In particular, the splitting, width, and relative intensities of the computed spectrum favorably agreed with the experimentally known properties. Based on this agreement, it was then also possible to assign the two spectroscopic signatures to distinct conformational substates. Those agreed with previous—more heuristic—attempts based on mutations in the active site and mixed quantum mechanics/molecular mechanics simulations based on a few representative snapshots from molecular dynamics simulations.^266,267^

##### b. 1 D- and 2 D-infrared spectroscopy of CN^–^

**FIG. 16.**
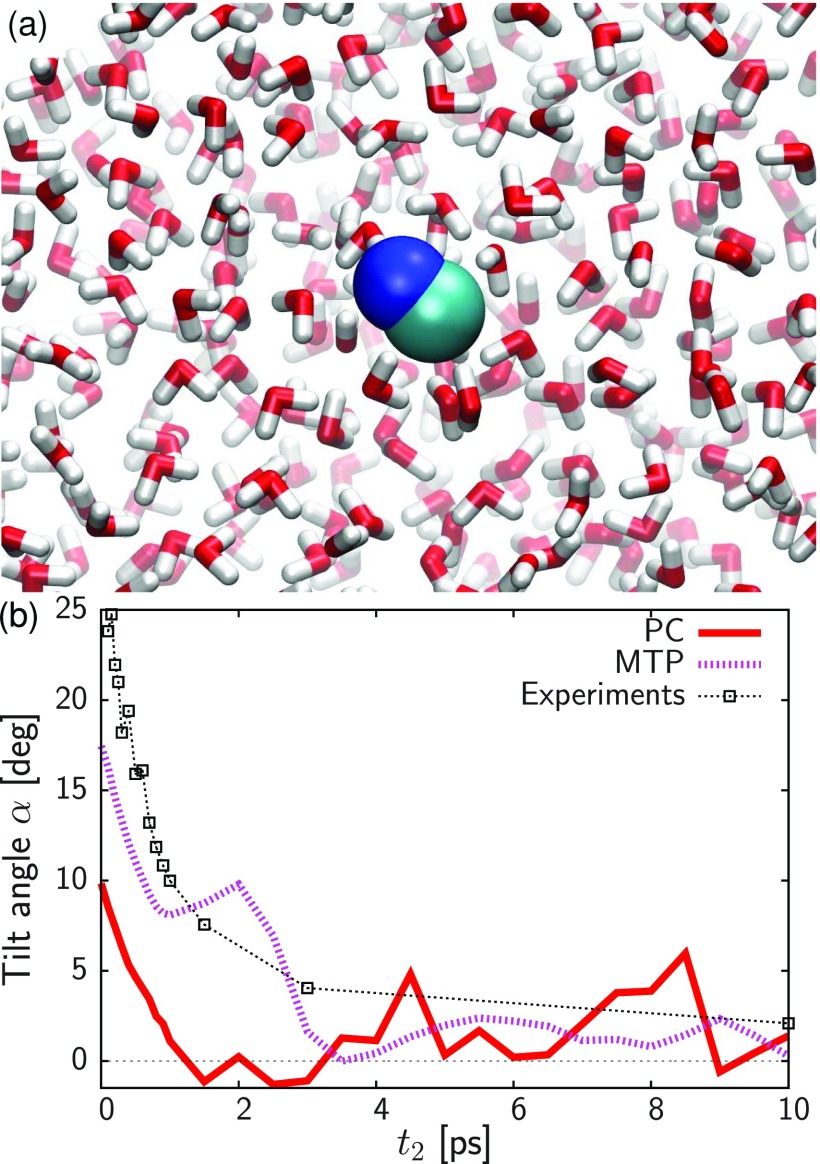
(a) Cartoon representation of cyanide in water. (b) Time evolution of the 2D-infrared tilt angle, *α*. The red, magenta, and blue curves correspond to PC, MTP, and experimental results, respectively. See Ref. 221 for more details.

The solution-phase spectroscopy of the cyanide anion (see Fig. 16) is another benchmark system for atomistic simulations as its dynamics has been studied extensively by experiments.^268–270^ The solution dynamics of small solute molecules provides detailed information on the coupling between intra- and intermolecular degrees of freedom. 2 D infrared spectroscopy has been shown to be sensitive to the solvent dynamics on the picosecond time scale which provides a benchmark to validate atomistic simulations against detailed experimental data.^271^

The 1 D- and 2 D-infrared spectrum of a hydrated probe can be determined from the frequency trajectory ω(t) from which the frequency-fluctuation correlation function C(t)=〈δω(0)δω(t)〉 can be determined. Here, δω(t)=ω(t)−〈ω〉 and 〈ω〉 is the average frequency of the oscillator along the trajectory. The correlation function contains time scales which are representative of the surrounding solvent motion and can be related to the experimentally measured spectral features. Those, in turn, are characterized by a tilt angle. Hence, following the frequency-fluctuation correlation function is directly related to following the spectral changes as a function of time in a 2 D-infrared experiment.^271^ Within a range of justifiable (and commonly used) force fields, one of the major experimental observables—the tilt angle *α* as a function of the waiting time—can be realistically modelled.^221^ Most importantly, an MTP model for water and cyanide combined with anharmonic stretching and bending potentials^272^ and slightly modified van der Waals ranges for the CN^–^ yields very favorable agreement with 2D infrared experiments.^270^ Conversely, a PC model misses almost all of the time dependence of the signal (see Fig. 16). Hence, MTP models provide a robust and realistic parametrization for dynamical problems including vibrational relaxation and 2D infrared spectroscopy.

It is also worth mentioning that an efficient and spectroscopically accurate force field for sampling the conformations obviates the need for specifically designing frequency maps in the computation of 2D infrared spectra. Such frequency maps are a convenient means to determine 2D infrared spectra from conventional molecular dynamics simulations.^273,274^ However, their transferability from one system to a chemically related one is not guaranteed, and they do not allow to carry out a consistent analysis of a physico-chemical process because conformational sampling and analysis (“scoring”) of the simulations employ different energy functions. In other words, only the use of a unique force field for both conformational sampling and post-processing allows us to uniquely trace back potential shortcomings of the energy function (e.g., CN^–^ in aqueous solution^221,272^).

##### c. Protein-ligand binding

The advantage of MTP over PC electrostatics coupled to a non-polarizable force field becomes evident when calculating the free energy of binding of a tetrabromobenzotriazole ligand with the target protein casein kinase 2:^275^ PC-only electrostatics have been shown to *destabilize* the complex,^276^ while the relative binding free energy between PC and MTP descriptions yielded a 3.8 kcal/mol increased stability though no absolute free energy calculation was reported.^20^

A recent application of refined electrostatic interactions in atomistic simulations concerned the *ab initio* determination of protein-ligand binding poses from computation combined with linear infrared experiments. Stark shifts can be used to study the structure, electrostatics, and dynamics of ligands and spectroscopic probes in protein active sites.^277–282^ The dynamics and spectroscopic response of chemical bonds to changes in the local electric fields can be accurately measured through 1-dimensional spectroscopy. However, relating spectroscopic information to changes in the structure of the environment surrounding the spectroscopic probe is not straightforward because simultaneous observation of spectroscopy and structure is still difficult.^283^ Atomistic simulations using validated force fields^189^ provide a valuable complement. The preferred use of physics-based empirical force fields^21,193,194,222,284^ over *ab initio* molecular dynamics simulations derives from the fact that comprehensive conformational sampling for a protein-ligand complex in solution is currently not possible due to the computational expense of *ab initio* molecular dynamics.

The nitrile group (-CN) is a meaningful spectroscopic label for probing the local structure, electric field, and solvent dynamics involving proteins and biological molecules.^278,279,285–288^ Previously used nitrile probes for proteins include CN-labelled phenylalanine^289–291^ and the nitrile-containing IDD type inhibitor for human aldose reductase.^278,292^ Benzonitrile (PhCN) is another potentially useful probe to determine the local electrostatic environment as it fulfills three important criteria: the -CN stretching mode at ≈2200 cm^–^^1^ (a) absorbs in a frequency range (i.e., between 1800 cm^–^^1^ and 2800 cm^–^^1^) in which proteins containing only naturally occurring amino acids have no vibrational spectral response (except for the -SH group in cysteine), (b) is to a good approximation a local mode (i.e., uncoupled from other framework modes), and (c) the dipole moment of PhCN is to a large extent that of the nitrile group itself. On the other hand, the nitrile group may pose additional challenges in concrete experiments due to its low extinction coefficient.^293^ The previous work on PhCN in water^294^ provides an ideal benchmark to validate the computational methods used in the present work.

Using a fluctuating point charge model for PhCN fitted to the molecular electrostatic potential, the dynamics of PhCN in the benzene-binding site of Lysozyme was investigated.^295^ The model was validated against 1d- and 2d-infrared experiments of PhCN in solution (water). Using the wild-type and two mutated proteins (L99A and L99G) which provide different electrostatic environments in the active site, the simulations find that the peak frequency of the -CN stretch in the linear absorption spectrum shifts. The shift approximately correlates with the relative binding free energy: the stronger the binding, the larger the red shift. This is a useful basis for the proposed strategy to locate ligand-binding sites through a combination of experiment and computation.^278^ The long time scale decay constant of the frequency-fluctuation correlation function is largest (2.0 ps) for the L99A mutant to which PhCN binds most strongly. Given that in state-of-the-art experiments a relaxation time can be determined to within 40%, the wild-type and L99G show a similar *τ*_2_ and the binding of PhCN to these two protein variants is weaker. Hence, strong protein-ligand binding correlates with long decay times in the frequency-fluctuation correlation function. Finally, a pronounced static inhomogeneous component ($\Delta_{s}^{2}=0.2$ ps^–^^1^) is found in the correlation function which appears, which is absent for PhCN in water. However, the magnitude of $\Delta_{s}^{2}$ does not appear to be related to the binding strength.

##### d. Vibrational Relaxation of Solvated CN^–^

The exchange of energy between different degrees of freedom in a condensed-phase system is of fundamental importance. Energy flow is required for processes ranging from chemical reactivity to signalling in biological systems. Direct determination of energy migration pathways in molecular systems from experiments alone is very challenging. Hence, atomistic simulations with dedicated force fields are a powerful complement.

Atomistic simulations have shown to give energy relaxation times in good agreement with experiments.^272,296^ It has been found that vibrational energy relaxation is particularly sensitive to the level at which the intermolecular interactions are described and that models beyond conventional point charges are required for quantitative computational work. This provides the basis for more detailed investigations of the spectroscopy of CN^–^ in D_2_O, specifically whether a *single* parametrization of the intermolecular interactions is capable of quantitatively describing a number of distinct experimental observables.

Infrared experiments were used to determine *T*_1_ relaxation times of the *v* = 1 state of CN^–^ in H_2_O and D_2_O.^269,297^ In contrast to polyatomic molecules such as $N_{3}^{-}$, energy relaxation in diatomics is governed by intermolecular interactions and the coupling between solvent and solute can be investigated directly. It has been suggested^297^ and later confirmed^269,296^ that Coulomb interactions are responsible for the vibrational relaxation of polar molecules in coordinating solvents, such as water. Therefore, atomistic simulations with accurate MTP electrostatics are expected to provide detailed insights into energy migration pathways. Many previous simulations were carried out with idealized interaction potentials. For example, rigid water models are unable to reproduce energy flow into the water's internal degrees of freedom.^296^

Simulations with fully flexible force fields and accurate representations of the nonbonded interactions for CN^–^ and H_2_O provide quantitative agreement with experimentally determined relaxation times.^272^ Using a rigid water model, energy relaxation from the vibrationally excited chromophore (CN^–^) into the surrounding solvent is slower by more than an order of magnitude. Hence, under the given circumstances (existence of mechanical resonances between chromophore vibrations and internal solvent degrees of freedom) and for this type of study, it is mandatory that atomistic simulations are carried out with fully flexible monomers. The simulations also show that the calculated *T*_1_ times sensitively depend on the force field parametrization, in particular the Lennard-Jones ranges. Increasing the Lennard–Jones ranges by up to 7.5% simulations leads to longer relaxation times by a factor of 4 to 5. This can be qualitatively understood by noting that for larger Lennard–Jones ranges the distance between the solvent water molecules and CN^–^ will be larger on average which, in turn, leads to reduced electrostatic interactions and hence less efficient vibrational energy transfer.

In summary, the work on hydrated CN^–^ highlights that with one and the same force field parametrization based on MTP electrostatics it is possible to accurately describe sub-ps (2 D-infrared), ps (2 D-infrared), 10-ps (vibrational relaxation), infrared, and thermodynamic observables.^221,272,298^ Therefore, physics-based force fields provide the necessary improvement and level of accuracy required to provide molecular-level insight into condensed-phase energetics and dynamics.

## IV. CONCLUSION

We have presented several approaches for describing ultrafast dynamics induced by the interaction of molecules with light. Rather than providing a comprehensive review of one area, we have chosen several representative examples of methodologies and applications from the fields of quantum, semiclassical, and classical dynamics. Ultimately, one would like to treat both electrons and nuclei quantum mechanically, yet, as we have seen, many interesting phenomena can be described accurately with mixed quantum-classical (as in the trajectory surface hopping implementation of the local control theory in Sec. III A 2), semiclassical (as in the thawed Gaussian approximation evaluation of various types of electronic spectra in Secs. III B 2 and III B 3), or classical dynamics (as in the 1 D- and 2 D-infrared spectroscopy of CN^–^ in Sec. III C 2). Where nuclear quantum effects are important, one should of course use quantum or semiclassical approaches, both of which are capable to include nuclear quantum coherence, zero point energy, and sometimes also tunneling effects. Regarding the treatment of electronic structure, we have presented both on-the-fly *ab initio* dynamics (quantum Bohmian dynamics in Sec. III A 1, mixed quantum-classical trajectory surface hopping in Sec. III A 2, semiclassical thawed Gaussian approximation in Secs. III B 2 and III B 3) and classical dynamics based on high-quality parametrized reactive and multipolar force fields (in Secs. III C 1 and III C 2). On one hand, the latter, highly efficient analytical force fields will clearly always be in demand for applications in the systems with the largest number of atoms. On the other hand, it appears that on-the-fly *ab initio* dynamics will become increasingly practical not only for classical but also for semiclassical and trajectory-based quantum molecular dynamics.

## NOMENCLATURE

ARMD: adiabatic reactive molecular dynamics
CASPT2: complete active space second-order perturbation theory
CCSD(T): coupled cluster with single, double and perturbative triple excitations
CDR: cellular dephasing representation
CDRP: cellular dephasing representation with a prefactor
DFT: density functional theory
DPT: double proton transfer
DR: dephasing representation
DRP: dephasing representation with a prefactor
EVB: empirical valance bond
FAD: formic acid dimer
GDR: Gaussian dephasing representation
LCT: local control theory
LR-TDDFT: linear response time-dependent density functional theory
MD: molecular dynamics
MTP: multipole
MP2: second-order Møller–Plesset perturbation theory
NABDY: nonadiabatic Bohmian dynamics
OTF-AI-TGA: on-the-fly *ab initio* thawed Gaussian approximation
PC: point charges
PhCN: benzonitrile
SDM: symmetric double minimum
SPF: single-particle function
SSM: symmetric single minimum
TDDFT: time-dependent density functional theory
TGA: thawed Gaussian approximation
TSH: trajectory surface hopping
4-HA: 4-hydroxyacridine
